# Compositional Bias in Naïve and Chemically-modified Phage-Displayed Libraries uncovered by Paired-end Deep Sequencing

**DOI:** 10.1038/s41598-018-19439-2

**Published:** 2018-01-19

**Authors:** Bifang He, Katrina F. Tjhung, Nicholas J. Bennett, Ying Chou, Andrea Rau, Jian Huang, Ratmir Derda

**Affiliations:** 1 Department of Chemistry and Alberta Glycomics Centre, University of Alberta, Edmonton, AB T6G 2G2 Canada; 2 Key Laboratory for NeuroInformation of Ministry of Education, School of Life Science and Technology, University of Electronic Science and Technology of China, Chengdu, 610054 China; 3 GABI, INRA, AgroParisTech, Université Paris-Saclay, 78350 Jouy-en-Josas, France; 4 Center for Information in Biology, University of Electronic Science and Technology of China, Chengdu, 610054 China; 5 The Scripps Research Institute, 10550 N. Torrey Pines Rd., La Jolla, CA 92037 USA; 6 The Salk Institute, 10010 N. Torrey Pines Rd., La Jolla, CA 92037 USA

## Abstract

Understanding the composition of a genetically-encoded (GE) library is instrumental to the success of ligand discovery. In this manuscript, we investigate the bias in GE-libraries of linear, macrocyclic and chemically post-translationally modified (cPTM) tetrapeptides displayed on the M13KE platform, which are produced via trinucleotide cassette synthesis (19 codons) and NNK-randomized codon. Differential enrichment of synthetic DNA {**S**}, ligated vector {**L**} (extension and ligation of synthetic DNA into the vector), naïve libraries {**N**} (transformation of the ligated vector into the bacteria followed by expression of the library for 4.5 hours to yield a “naïve” library), and libraries chemically modified by aldehyde ligation and cysteine macrocyclization {**M**} characterized by paired-end deep sequencing, detected a significant drop in diversity in {**L**} → {**N**}, but only a minor compositional difference in {**S**} → {**L**} and {**N**} → {**M**}. Libraries expressed at the N-terminus of phage protein pIII censored positively charged amino acids Arg and Lys; libraries expressed between pIII domains N1 and N2 overcame Arg/Lys-censorship but introduced new bias towards Gly and Ser. Interrogation of biases arising from cPTM by aldehyde ligation and cysteine macrocyclization unveiled censorship of sequences with Ser/Phe. Analogous analysis can be used to explore library diversity in new display platforms and optimize cPTM of these libraries.

## Introduction

Display platforms, such as phage display, yeast display and mRNA display, are the workhorses for the development of biological drugs^1,2^ and the identification of peptide and peptide derivative candidates for applications in diagnostics, therapeutics and drug-delivery^3^ and biomaterials and inorganic functional materials^4,5^. Although original display systems were limited to the 20 canonical amino acids, introduction of chemical post-translational modifications (cPTM) into phage and mRNA-libraries dramatically expanded the diversity of these libraries^6–9^. Selection from genetically-encoded (GE) cPTM-libraries provides access to chemically-modified peptide ligands with added benefits such as constrained topology^10^, proteolytic stability^8^, glycosylation^11,12^, etc. Development of efficient search strategies for the discovery of functional ligands from a library of displayed polypeptides, or their derivatives, requires knowledge of the exact composition of the naïve libraries from which they are sourced. Foundational work that spans multiple fields from RNA libraries^13^ and SELEX^14^ to phage-displayed peptide libraries^15^ teaches that the composition of a mixture of molecular entities, such as DNA, RNA and phage, changes when these entities compete for the same resources, such as polymerase or a host that replicates the phage. Library-wide assessment of chemical modifications is presently a challenging task; existing methods reveal the overall yields of reactions but cannot pinpoint whether sub-sets of library members have preferential or decreased reactivity towards modification. Understanding these biases, in turn, is important because skewed composition of the naïve GE and GE-cPTM libraries influences the outcome of the selection of ligands from these libraries.

Biases can be broadly categorized as “censored” and “parasite” sequences. In phage display, censorship of charged sequences through the Sec pathway is well known^16^. Dower and co-workers demonstrated that phage clones that displayed Arg-rich sequences decreased production by a factor of 10^2^–10^6^ and that this censorship scaled exponentially with the number of Arg residues in the sequence^16^. Makowski and colleagues subsequently observed the same censorship in commercially available phage displayed libraries of peptides (Ph.D.-12 and Ph.D.-C7C libraries) by Sanger sequencing of ~100 clones^17^. Makowski also predicted censorship of α-helix or β-sheet conformations in the same phage-displayed libraries^17^. Plückthun and others, in turn, have shown experimentally that folded proteins do not export well via Sec pathway^18^. The opposite, “parasite” sequences have been characterized by multiple groups as clones within naïve libraries that have mutations in distal regulatory regions in the phage genome, which afford amplification advantages^19–23^. Roberts and co-workers have studied similar amplification biases present in mRNA-displayed libraries and postulated that these libraries contain both highly-expressed parasite-like and average level of expression subsets of sequences^24^. Preferential amplification of “parasite RNA sequences” was observed more than 20 years ago by Breaker and Joyce^13^. Since this report, multiple mechanisms of enrichment and suppression of sequences in oligonucleotide libraries have been described^14,25–27^. The replication-suppressed “censored” and replication-enhanced “parasite” subsets, thus, are ubiquitously present in GE libraries.

Deep sequencing is now routinely used to evaluate the output of selections from phage display and other display constructs^12,28–38^. However, the characterization of naïve libraries by deep-sequencing has not been extensively conducted and is difficult for several reasons: (i) naïve libraries cover a large sequence space and thus contain a large number of low-copy-number reads; (ii) if libraries are constructed to have complete sequence coverage, then every sequence in such a library would be within Hamming distance (h = 1) of several sequences in the same library. (*The Hamming distance between two strings of equal length is the number of positions where the corresponding symbols are different: For example, two sequences GATTACA and CATTACA are separated by one substitution, and the Hamming distance between them is 1, h = 1*). Unfortunately, point mutations caused by sequencing and PCR errors give rise to sequences within h = 1 and these mistakes cannot be distinguished from true reads; (iii) systematic biases imposed by improper sequencing methods or analysis can lead to the severe inflation or deflation of library diversity^39^. Similar problems have often been encountered in the evaluation of metagenomes and bacterial populations^40^. The concept of a “species” in metagenomic analysis is similar to the concept of “unique sequences” in peptide libraries. For example, the number of “species” reported in early metagenomic studies was grossly overestimated, compared to when the same samples were re-sequenced and analyzed with higher scrutiny and fidelity^41^. Techniques such as Primer ID^42^ have been developed in metagenomic studies to detect false “species” (i.e., sequence differences) that arise from errors in sample preparation and processing. Here, we applied the same technique in the analysis of phage libraries.

The majority of display platforms operate with libraries of 10^9^ or higher diversity, but this level of diversity cannot be effectively covered using currently available sequencing tools. In this report, we produced three types of small tetrapeptide libraries that have a maximum diversity of 10^5^–10^6^, which is easily accessible to routine Illumina sequencing and data processing: (i) an N-terminally displayed library with the sequence Ser-X-Cys-XXX-Cys, where X is any one of 19 trinucleotide codons (“allowed” codons, cysteine excluded), termed NT-TriNuc^43^; (ii) an N-terminally displayed library encoded by conventional NNK randomization with the sequence Ser-XXXX, termed NT-SX4^44^; (iii) a library encoded by NNK randomization and expressed between N1 and N2 domains of phage coat protein pIII, termed ID-SX4^45^. We also developed a paired-end processing program tailored for the analysis of phage-displayed libraries. NT-TriNuc and NT-SX4 libraries were characterized by paired-end deep sequencing at every step of library production. Although paired-end processing pipelines are common in the literature, we sought to build on published concepts^22,46^ and re-implement the processing as an open-source MATLAB code. To track mutations caused by PCR, we used the Primer ID technology on the synthetic NT-TriNuc library. In addition, we also characterized biases arising from chemical post-translational modification (cPTM) through N-terminal oxime ligation^12,47^ and dichloro-oxime-mediated cyclization^11,12^.

## Results

### Overview and evaluation of paired-end processing

**Figure 1:**
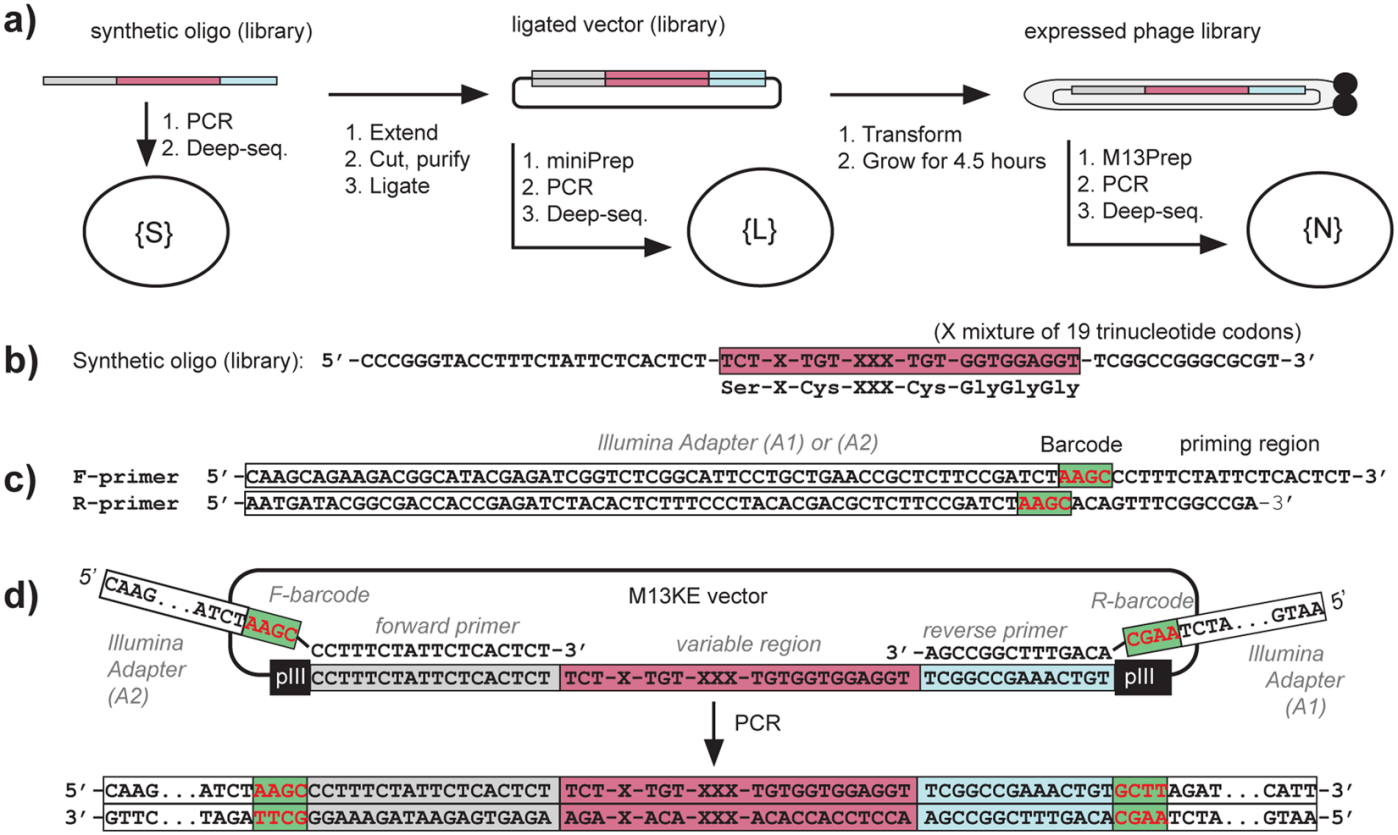
Analysis of diversity in naïve libraries. (**a**) The libraries were sequenced at every step of standard production of phage libraries: (i) synthesis of random oligonucleotide (“oligo”); (ii) extension and ligation into the vector; (iii) transformation into bacteria and expression of the library for 4.5 hours. After extension and ligation of synthesized oligonucleotides into the vector, the library was sequenced before the transformation into bacteria. (**b**) Synthetic NT-TriNuc library. (**c**) Primers used for amplifying ligated or naïve oligonucleotide DNA. (**d**) Generation of PCR product. Alignment of forward and reverse primers to 18-bp and 14-bp sequences flanking the variable region at the N-terminus of the pIII gene in M13KE vector, respectively.

High quality paired-end deep sequencing and analysis is critical for the evaluation of phage libraries at every step of their production (Fig. 1a). An overview of the paired-end processing routines and analysis of the effectiveness of these routines can be found in^48^ and references cited within. Many of the reports suggest that the quality score of reads provided by sequencing instruments can be inflated and thus, it is not clear that quality scores should be used as the sole metric to evaluate the accuracy of the reads. As an alternative to quality filtering, alignment of the reads in the constant regions before and after the variable region can be used as an evaluation criterion. The paired-end processing serves several purposes: (i) it increases the confidence of the read by covering the read region twice; (ii) it can be translated to longer libraries without any change in sequencing or analysis routine; (iii) it allows double indexing of the read using two independent sequencing barcodes. For example, we sequence 200 diverse samples simultaneously using only 10 forward and 20 reverse primers, whereas encoding 200 samples via single-end sequencing requires 200 distinct primers each with a distinct single index^46^. To select optimal processing parameters for paired-end sequencing, we analyzed a synthetic DNA library generated using a mixture of 19 “allowed” trinucleotide codons. Since this library is synthesized using a mixture of 19 defined trimer phosphoramidites, the presence of the other 45 codons (“forbidden” codons, FC) in this library can be attributed to mistakes that originate in DNA synthesis or the sequencing procedure.

**Figure 2:**
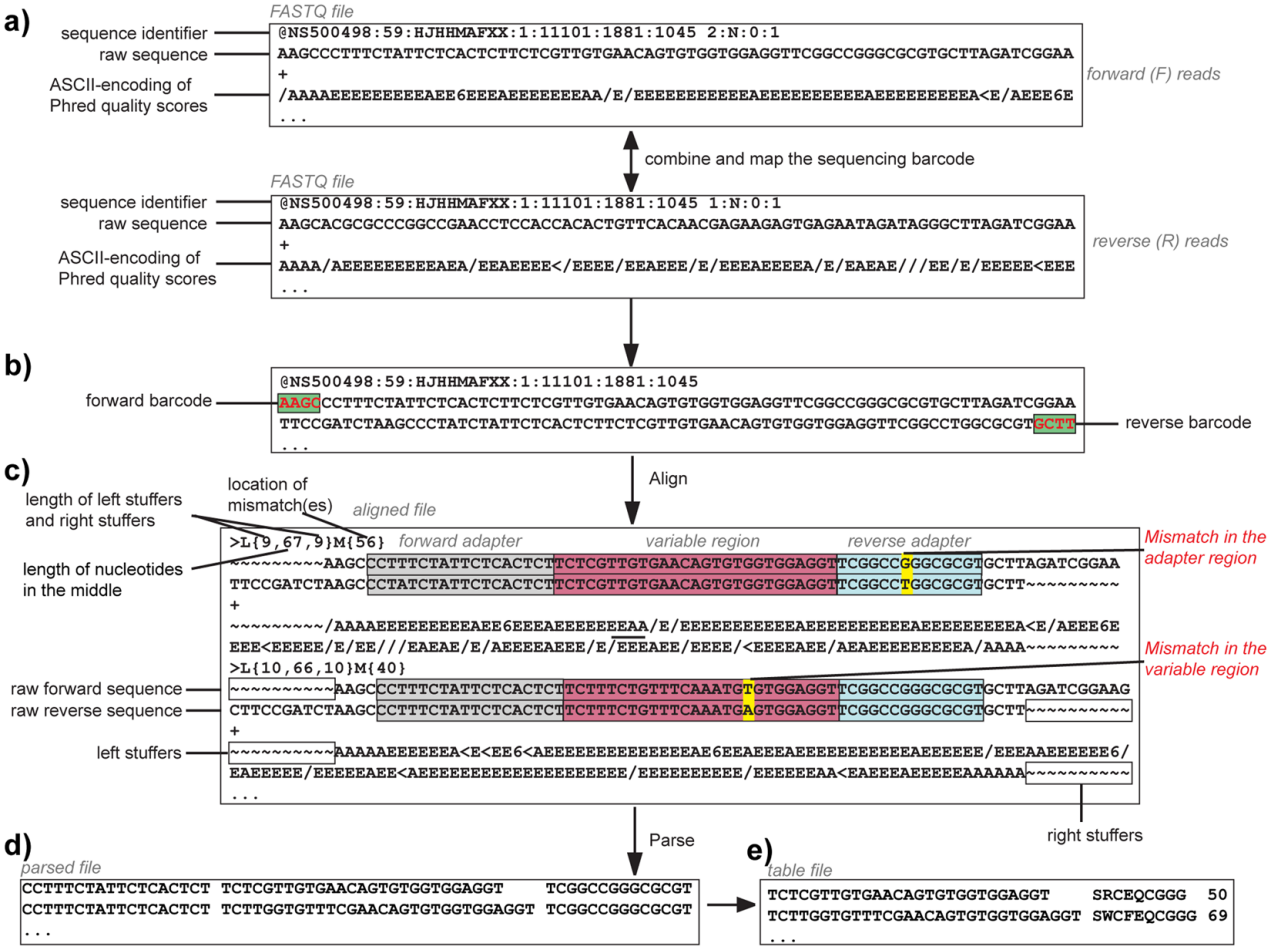
Workflow of the paired-end processing pipeline. The MATLAB script converts FASTQ files to the final table files via several steps: (**a**) combining F and R reads and mapping of sequencing barcodes; (**b**) tiling alignment of F and R FASTQ files to yield a FASTQ-like aligned format; (**c**) Addition of F + R reads. (**d**) parsing to match FA and RA sequences, permitting one mutation in each of the FA or RA regions; discarding reads with FR-mismatches in the library region; (**e**) translating the library reads and converting to a frequency table. A FASTQ file (in **a**) has four lines for each sequence: Line 1 begins with a ‘@’ character and is followed by a sequence identifier and an optional description; Line 2 is the raw sequence letters; Line 3 begins with a ‘ + ’ character and is optionally followed by the same sequence identifier (and any description) again; Line 4 encodes the quality values for the sequence in Line 2, and must contain the same number of symbols as letters in the sequence. “/AAAAEEEE.” (in **a**) is part of the standard FASTQ-format for ASCII-encoding of Phred quality scores. “Stuffers” (in **c**) are added symbols to find an optimal alignment between the F and R reads.

**Figure 3:**
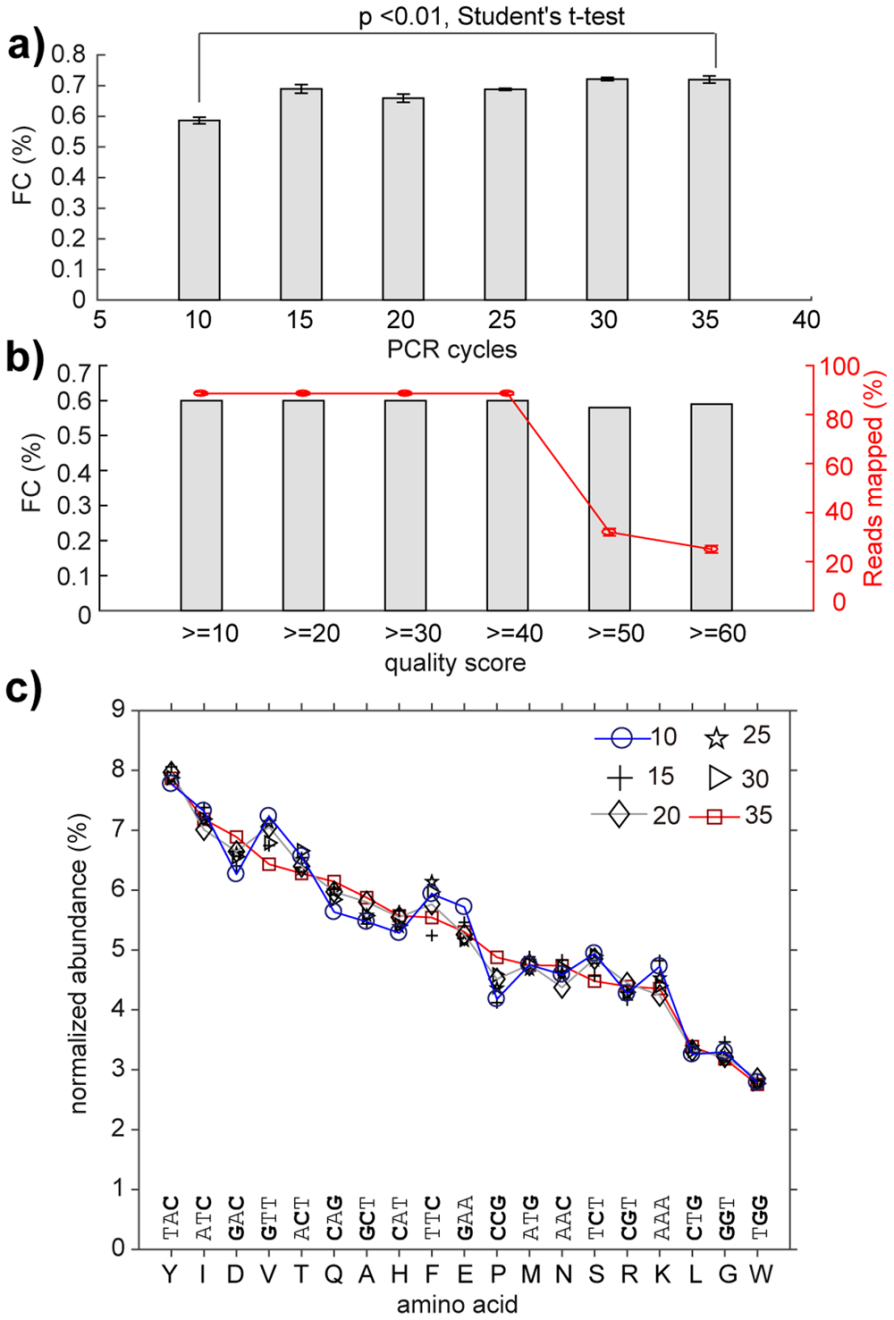
Effect of PCR parameters on quality of deep sequencing. (**a**) Increasing the number of PCR cycles from 10 to 35 led to only subtle changes in percentage (0.5% to 0.7%) of forbidden codons (FC). (**b**) The percentage of FC did not change significantly when reads were filtered by quality scores, but the overall fraction of mapped reads decreased dramatically when over-stringent quality filtering was employed. (**c**) We observed minor drift in the relative ratio of each TriNuc codon in all four positions of the synthetic libraries of Ser-X-Cys-XXX-Cys peptides when the number of PCR cycles was increased from 10 to 35.

Applying a previously published single-end analysis to process Illumina data originating from sequencing of synthetic DNA library^22,46^ yielded 99.25% “allowed” codons and 0.75% FC. The paired-end analysis (Fig. 2) of the same library yielded 0.58% FC. A fraction of the forbidden codons within the sequencing results, thus, was derived from discrepancy between the forward (F) and reverse (R) reads that resulted in mismatches between the F and R reading of the same sequence (FR-mismatches). The remaining FC could originate from the conversion of the single-stranded DNA (ssDNA) to Illumina-compatible double-stranded DNA (dsDNA) using PCR, or during the bridge amplification PCR used in Illumina sequencing. Although we employed one of the highest fidelity polymerases currently available (Phusion® High-Fidelity DNA Polymerase, New England Biolabs), there is still an inherent error rate in our pre-processing. However, reprocessing the synthetic DNA library by 10–35 cycles of PCR and Illumina analysis found a constant level of 0.5–0.7% FC regardless of the number of PCR cycles (Fig. 3a). No major drift for abundance of individual sequences was observed at different PCR cycles (Figure S1). We then employed Primer ID technology to further track and correct mutations introduced by the PCR step^42^. In Primer ID, a primer containing a barcode composed of 8 randomized nucleotides (65,536 distinct combinations) is used for a single-pass extension of each individual DNA molecule (Figure S2a). If subsequent PCR produces point mutations, the errors caused by PCR are manifested as point mutants with the same Primer ID barcode. This analysis (Figure S2b), however, found that only 11 out of ~30,000 reads with FC could be corrected by Primer ID methodology (Table S1). The combined results strongly suggest that pre-processing of reads by PCR is not a major source of the FC. The FC may originate either in the Illumina bridge amplification step or sequencing by synthesis and base-calling (Bases are identified from light intensity signals, a process commonly known as base-calling)^49^.

The most abundant FC were within Hamming distance h = 1 of multiple “true” codons (Figure S3g). However, neither the number of neighbors in Hamming-space nor the nature of mutation (transition vs. transversion) could unambiguously explain the abundance of all FC (e.g., Figure S3h), suggesting, again, that FC appear due to multiple mechanisms (e.g. on-chip bridge amplification and DNA synthesis during sequencing). The incorporation of additional filtering by Phred quality scores QuaF >  = Qcutoff & QuaR >  = Qcutoff for all bases in the library region after matching FA and RA sequences also had no significant effect on the percentage of FC (Fig. 3b). This additional quality filtering, however, dramatically reduced the number of reads available for further processing. These observations are consistent with our previous report in which we observed that in reads with matched adapter sequences, the correlation between Phred quality score and sequence accuracy is only modest^50^. We concluded that Q-filtering, thus could lead to false negatives and did not use this filtering in this report.

The above results suggest that no post-sequencing processing can completely remove FC, and while it is tempting to simply discard FC, such discarding can be justified only for synthetic, minimally handled libraries (i.e. samples that underwent no biological manipulation prior to sequencing). For analysis of naïve and selected libraries, it is necessary to retain FC, as the increase in fraction of FC might indicate mutations from the replication processes involved in the amplification of the phage in bacteria.

All of the above analyses were obtained using NextSeq® 500/550 Mid Output Kit v2 (150 cycles) (catalog # FC-404-2001). We found that sequencing data collected using NextSeq® 500 Mid Output Kit (150 cycles) (catalog # FC-404-1001) (prior to June 2015) contained a higher number of FC and FR-mismatches. Data summarized in Figures S3–S10 only partially identify the mechanism of these site-specific FR-mismatches. We have noted that these problems largely disappear upon introduction of the v2 kit in 2015.

### Evaluation of the loss of diversity in expression of the libraries

We monitored the sequence composition of our libraries at every step of the production: (i) synthesis of random oligonucleotide (“oligo”) to yield a synthetic library {**S**}; (ii) extension and ligation into the vector to yield a ligated library {**L**}; (iii) transformation of the ligated vector into the bacteria followed by expression of the library for 4.5 hours to yield a “naïve” library {**N**} (Fig. 1a). PCR appended the adapters required for Illumina sequencing (Fig. 1b). The reverse PCR primers R1 described in Fig. 1c amplified only sequences that were ligated into the M13KE vector, while reverse primer R2 shown in Figure S11 only amplified synthetic DNA that was not cut by EagI restriction enzyme. These primers, thus permitted independent analysis of the {**S**} and {**L**} DNA subsets in a mixed population of synthetic, unligated and ligated DNA in step (ii).

**Figure 4:**
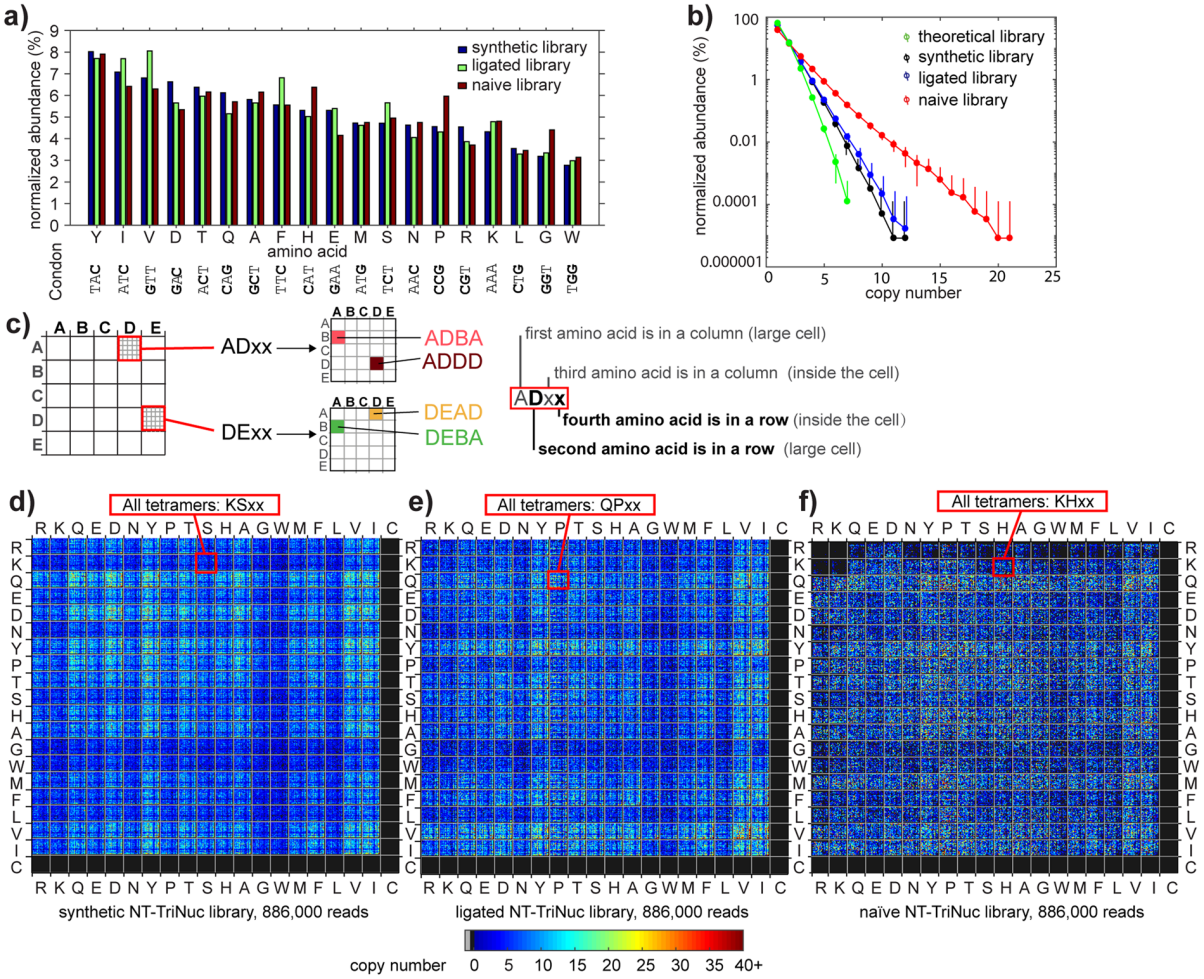
Composition of NT-TriNuc library before and after expression. (**a**) Distribution of TriNuc codons in all four positions of the synthetic, ligated and naïve Ser-X-Cys-XXX-Cys tetrapeptide libraries. (**b**) The distribution of copy numbers in the libraries was calculated using random samples of 60,000 reads from the library. Error bars are standard deviations. For reference, we used random libraries of 60,000 peptides with a uniform ratio of 19 amino acids. (**c**) Description of a plot showing each peptide in the library as a unique pixel in a specific location. A model 5 × 5 letter plot with 25 × 25 = 625 pixels describes the location of common 4-letter words that contain the letters A, B, C, D and E. (**d**–**f**) In the 20:20 plot, the top left quadrant has all peptides of sequence RRxx, top right: RCxx; bottom left: CRxx and bottom right: CCxx. Color indicates the copy number. Each plot contains a random sample of 886,000 reads from deep sequencing for synthetic, ligated and naïve libraries (this number represents ~7 × coverage of theoretical peptide diversity (19^4^ = 1.3 × 10^5^)).

Sequencing of the synthetic library showed that the codon composition of the NT-TriNuc libraries deviates from a perfect 1:1:1.. ratio (Figs 3b and 4a). As the ratio did not change as a function of PCR cycles (Fig. 3b) and only weakly depended on the GC/AT ratio in codons, we attributed this to biases in DNA synthesis. After ligation and cloning of this library into the M13KE vector, the amino acid distribution was further skewed (Fig. 4a). In the expressed library, there is a selection against sequences containing Arg (Fig. 4a). This is similar to the bias that was characterized by Dower^16^ and Makowski using low-throughput Sanger sequencing^17^. When we examined the positional dependence of amino acids, the majority of this bias was in the penultimate N-terminal amino acid of Ser-X-Cys-XXX-Cys (Figure S12).

Deep sequencing illuminated the changes in the library-wide composition during production or selection. One of the challenges, however, was effective visualization of these changes within 10^5^–10^6^ sequences at once. To achieve this visualization, we employed a “20:20 plot” introduced in our previous publication^51^, which assigns a unique position on a 20 × 20 grid to every peptide sequence in the library based on the 1^st^ and 2^nd^ amino acid in that peptide (Fig. 4c). The plot is a fractal graph: “zooming in” on each element of the 20:20 plot reveals another 20:20 plot based on the 3^rd^ and 4^th^ amino acid. While we use it to map tetramer peptides, the 20:20 plot can be applied to a library of 2N-mers with N-levels of “zoom-in”. Visualization of all sequences in the expressed naïve phage library using a 20:20 plot suggested a strong bias against sequences that contain two or more positive charges (Fig. 4c–f). A minor sub-set of sequences corresponding to 0.01% of the library reached an unusually high copy number (copy number > 10) that had not been observed in the synthetic or ligated libraries (Fig. 4b). Unlike sequence-specific censored peptides clustered in one location of the 20:20 plot, the enriched sequences exhibited no sequence similarity as they were uniformly distributed on the 20:20 plot.

**Figure 5:**
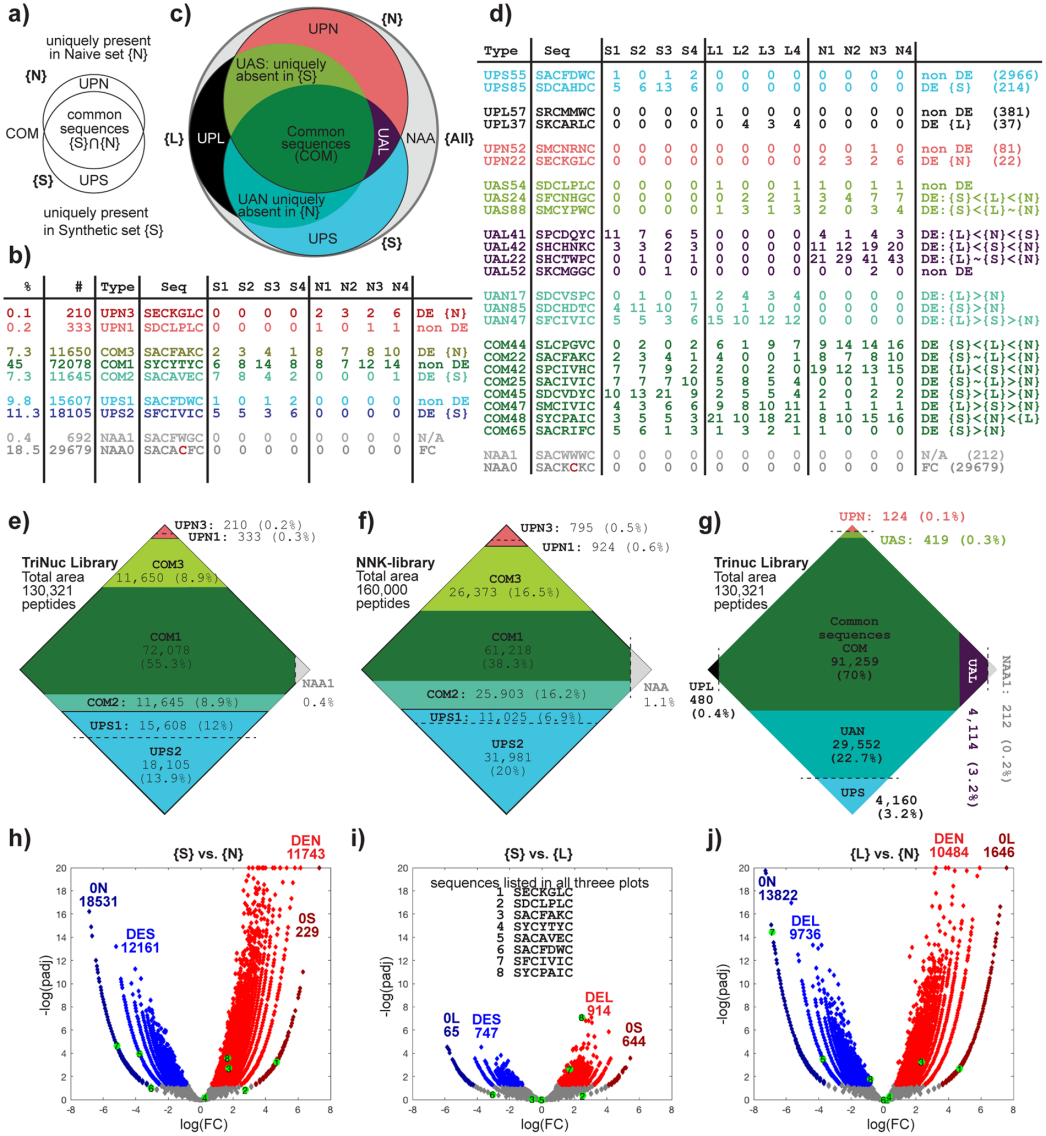
Differential enrichment analysis of NT-TriNuc and NT-SX4 libraries. (**a**) Venn Diagram comparison of Naïve {**N**} and Synthetic {**S**} sets and definitions of common (**COM**) and uniquely present sequences (**UPS**, **UPN**). (**b**) Table of sequences and their copy numbers observed in sequencing highlights that sequences from **COM**, **UPS**, **UPN** subsets can be differentially enriched (DE) and non-DE. (**c**) Comparison of {**S**}, {**N**} and ligated {**L**} sets, and definition of “uniquely absent” sets (**UAS**, **UAL** and **UAN**). (**d**) Example of reads, their copy numbers and their classifications; there exist several DE-classes (see Figure S13 for further classification). (**e**) To-scale representation of the entire NT-TriNuc library, in which the area of each segment is proportional to the number of unique sequences in each type listed in (**b**). For example, there are 95,373 (**COM1**: 72,078, **COM2**: 11,645, **COM3**: 11,650) unique sequences present both in {**S**} and {**N**} sets. (**f**) Analogous description of NT-NNK library. 38% of the library contains sequences that are neither significantly enriched nor depleted between {**S**} and {**N**} (COM1: 38.3%). About 27% of library sequences are uniquely present in {**S**} (**UPS1**: 6.9% and **UPS2**: 20%). (**g**) Analogous to-scale representation of an overlay of {**S**} and {**L**} and {**N**} from NT-TriNuc library shows that of 26% of sequences identified as **UPN** in (**e**), 22% are present in both {**S**} and {**L**} (i.e., “Uniquely Absent from Naïve” or UAN) and only 3.2% and 0.4% are unique to {**S**} or {**L**}. Note that in the **COM** set in (**g**), DE-information is omitted for clarity. (**h**–**j**) Volcano plots describing DE-comparison of {**S**}, {**N**} and {**L**}. {**S**} and {**L**} are most similar to one another whereas {**N**} is different from both {**S**} and {**L**}. The sequences listed in (**b**) are mapped on each volcano plot. Abbreviations: **DES** – differentially enriched in synthetic, **DEL** – differentially enriched in ligated, **DEN** – differentially enriched in naïve, **0S** – not present in synthetic, **0L** – not present in ligated, **0N**– not present in naïve. For details and algorithm for DE-analysis see R.zip file in the Supplementary Information for *RMD R-code. A detailed description of the terms is available in Figure S16.

Although 73% of unique sequences are common between {**S**} and {**N**} sets of the NT-TriNuc libraries (Fig. 5a,b), differential enrichment (DE) analysis detected that 9% of peptides were either enriched or depleted in the {**S**} → {**N**} transition, leaving only 55% as non-DE (Fig. 5b,e). DE was quantified using a negative binomial model with TMM–normalization and Benjamini–Hochberg (BH) correction to control the false discovery rate at α = 0.05 (see R.ZIP in the Supplementary Information for detailed statistics and R-code)^52^. Comparison of {**S**}, {**L**} and {**N**} sets shows that a large fraction of these sequences (22%) were present in {**S**}, {**L**} but not {**N**}; hence the major loss occurred during expression of ligated vector ({**L**} → {**N**} transition). A small loss (3%) also occurs during the ligation ({**S**} → {**L**} transition), resulting in sequences that are uniquely present only in the {**S**} set. DE of pair-wise comparison of {**S**} and {**N**} sets can be visualized via volcano plot (Fig. 5h) or segment diagram (Fig. 5e) in which the area of each segment is proportional to each DE-subset. DE of {**S**}-{**N**}, {**S**}-{**L**} and {**L**}-{**N**} sets confirm an observation described in 20:20 plots (Fig. 4d–f): the majority of the loss of diversity observed in {**S**} → {**N**} transition takes place in {**L**} → {**N**} transition, whereas differences in the {**S**} → {**L**} transition are minor and there are fewer DE-sequences. Many sequences depleted in the {**S**} → {**L**} transition contained KpnI restriction sites within the library region and, thus, were incompatible with the cloning process. Sequences differentially depleted in the {**L**} → {**N**} transition exhibited amino acid compositional biases such as depression of Arg^17,31^. A few sequences and their classification are described in Fig. 5d. A complete list of sequences and their classification are available as part of the Supporting Information (See Naïve_Libraries.ZIP).

**Figure 6:**
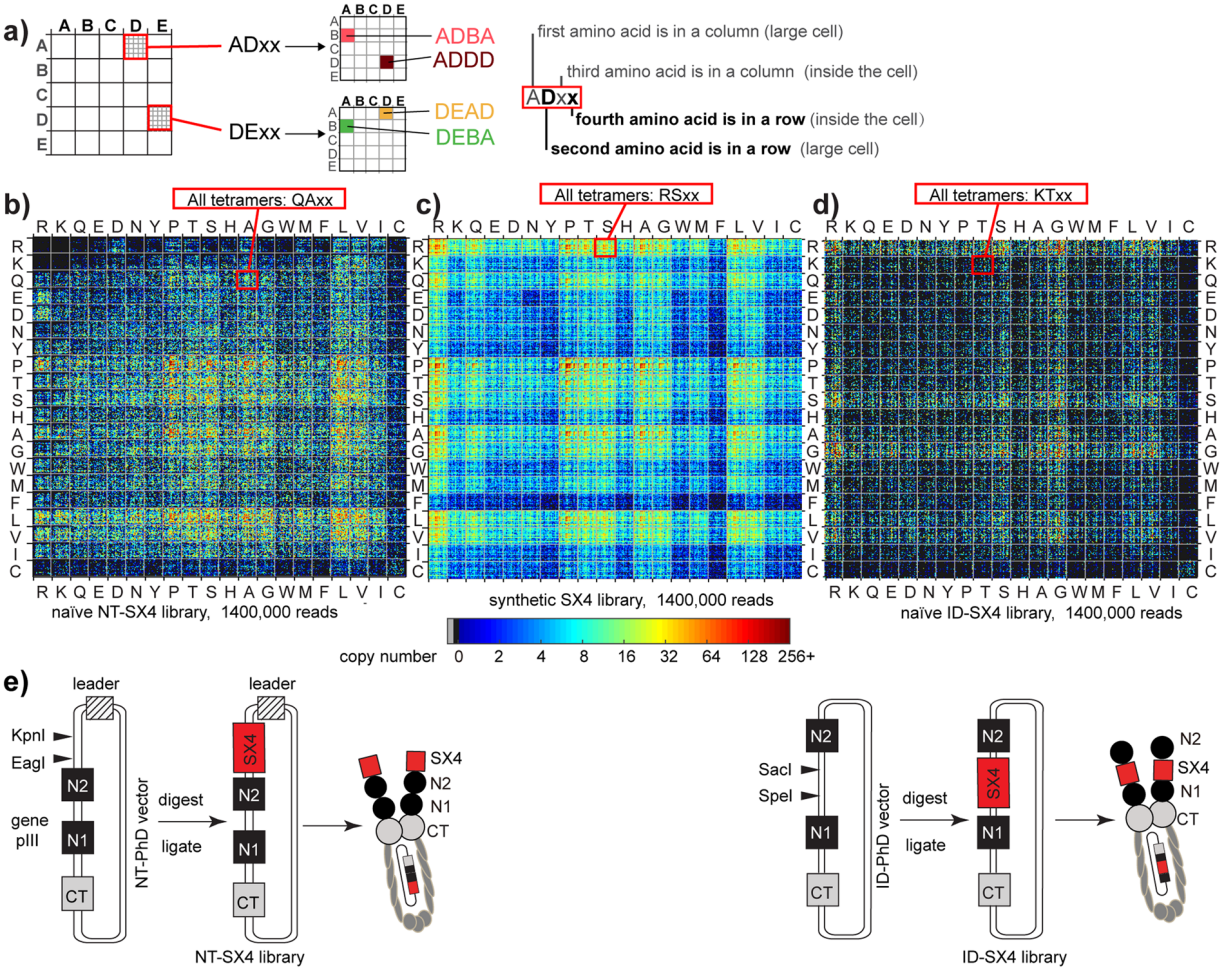
Diversity of libraries cloned in different locations of phage capsid. (**a**) Description of a plot describing each peptide in the library as a unique pixel in a specific location. A model 5 × 5 letter plot with 25 × 25 = 625 pixels describes the location of common 4-letter words that contain the letters A, B, C, D and E. (**b**–**d**) The 20:20 plot, where color indicates the copy number and each plot contains a random sample of 1.4 million reads from deep sequencing of (**b**) naïve NT-SX4 libraries, (**c**) synthetic SX4 libraries and (**d**) naïve ID-SX4 libraries. In 20:20 plot, as in 5 × 5 plot in panel (**a**), each tetrapeptide is represented by a unique pixel in a specific location. The top left quadrant has all peptides of sequence RRxx, top right: RCxx; bottom left: CRxx and bottom right: CCxx. The number of reads sampled (1.4 × 10^6^) represents 0.08 × coverage of theoretical nucleotide sequence diversity (4^14^ = 1.67 × 10^7^) and ~10 fold average coverage of theoretical peptide diversity (20^4^ = 1.6 × 10^5^). (**e**) Schematic comparison of NT-SX4 and ID-SX4 library production. In the NT-SX4 library (left), the SX4 library is expressed at the N-terminus of the N1 domain of pIII, while in the ID-SX4 library (right), the same library is expressed between N1 and N2 domains of pIII.

Analysis of the NT-SX4 library, in which X is a random amino acid encoded by NNK codon, can be performed similarly. The non-uniform abundance of amino acids required an increased depth of sequencing for analysis and made direct comparison of NT-SX4 and NT-TriNuc libraries challenging. For example, sample size used in the 20:20 plot in Fig. 4d–f provides 7-fold coverage of 19^4^ diversity in NT-TriNuc library. If peptide diversity in NT-SX4 library is crudely estimated as 20^4^, a sample size of 1.3 million sequences produces a 20:20 plot at 9-fold coverage of this diversity. We note that while these calculations are not optimal, such estimate of diversity and such sample sizes are frequently used in experiments that use NNK-encoded libraries. A comparison of 20:20 plots of {**S**} and {**N**} sets of NT-SX4 libraries also shows that the composition of the NT-SX4 library changes dramatically in the {**S**} → {**N**} transition (Fig. 6b,c), and that the majority of this loss takes place in {**L**} → {**N**} transition (Figure S14). In addition to censorship against Arg and Lys sequences, Makowski and co-workers^17^, predicted existence of library-wide censorship of Cys in NNK libraries. Indeed, comparing 20:20 plots of {**S**} and {**N**} NNK-libraries confirms significant depletion of all Cys-containing sequences (Fig. 6b,c). Interestingly, while the NNK-encoding scheme was significantly different from NT-TriNuc (comparing Figs 4f and 6b), the losses of unique sequences during the {**S**} → {**N**} transition were similar between NT-SX4 and NT-TriNuc libraries (Figure S15). For example 26% and 27% of synthetic sequences were not present in naïve NT-SX4 and NT-TriNuc libraries, respectively. The similarity in biases between these two libraries emphasizes that the majority of the {**S**} → {**N**} bias is driven by peptide sequence. We noted an increased fraction of differentially enriched or depleted sequences in NT-SX4 when compared to NT-TriNuc. This increase might originate from non-uniform distribution of peptides in NNK-encoded libraries arising from NNK codon usage^53^.

### Comparison of N-terminal and intra-domain libraries

To overcome the bias against positively charged peptide sequences, we hypothesized in our previous report^45^ that expressing libraries at a different location of pIII, between N1 and N2 domains instead of at the N-terminus of the N1 domain, would minimize this bias. The scheme comparing the NT-SX4 and ID-SX4 libraries are shown in Fig. 6e. Sequencing the naïve ID-SX4 library revealed that indeed, the biases against positively-charged sequences observed in NT-SX4 library were alleviated in ID-SX4 library (Fig. 6b–d). Unfortunately, the ID-SX4 library exhibited a drastic bias towards Gly- and Ser- rich sequences and a strong censorship of peptides with Pro, Thr, Leu and Val (Fig. 6d). As the linker connecting N1 and N2 regions is rich in Ser and Gly, this bias towards Gly or Ser, but not closely related Thr, is not entirely surprising. To our knowledge, this is the first investigation of amino-acid preference of linker composition, uncovering an uncharacterized role of intra-domain linkages in effective production of phage.

### Collapse of diversity and censorship in chemical post-translationally modified libraries

**Figure 7:**
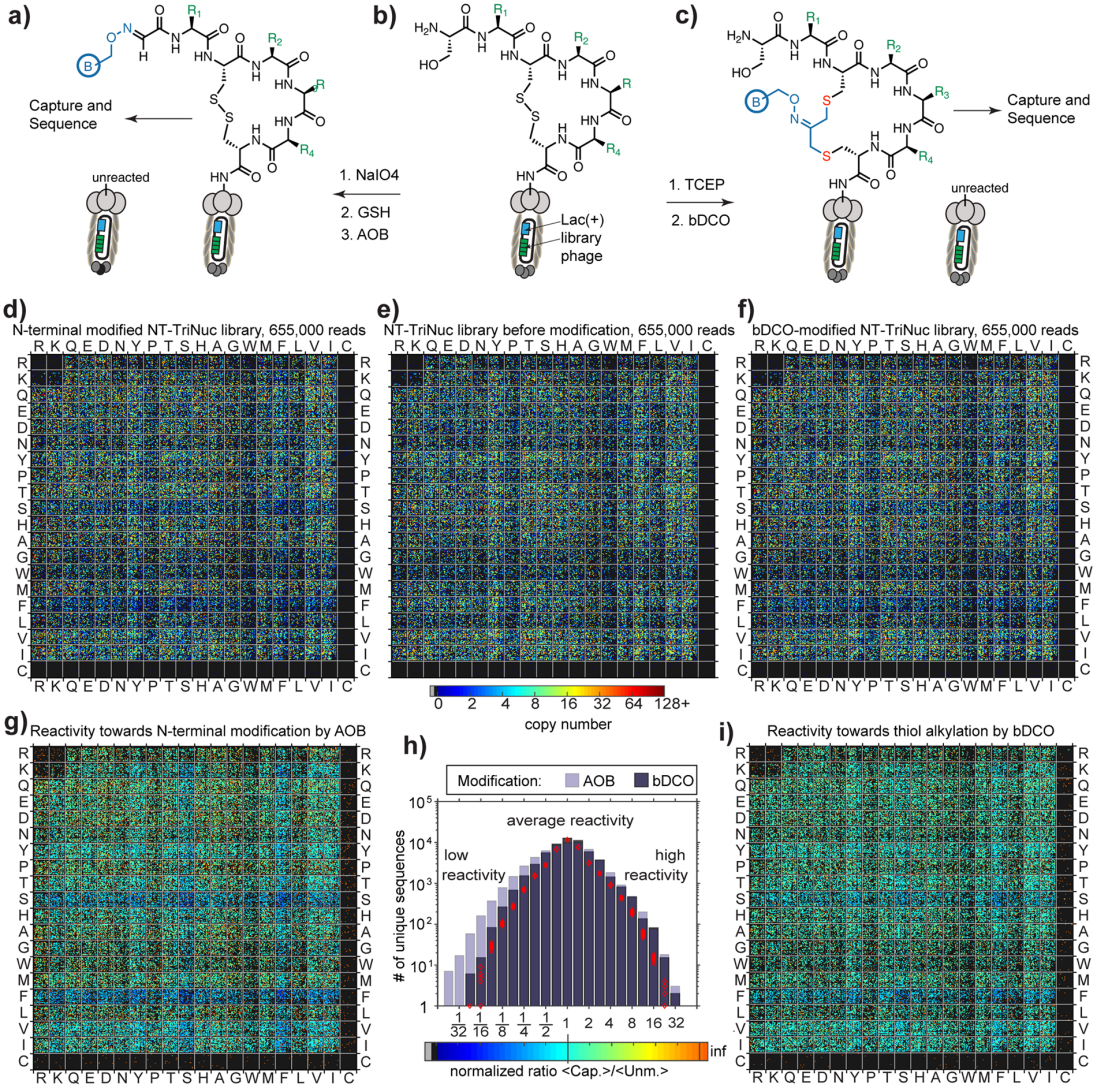
Diversity of libraries before and after chemical modification. (**a**–**c**) Workflow of chemical modification of the NT-TriNuc library. (**d**–**f**) Comparison of the peptide sequence composition of libraries before (**e**) and after chemical modification by AOB (**d**) or bDCO (**e**) and capture of the reacted populations. Compared to the library before modification, an interesting lack of Phe/Ser-containing peptides was observed in chemically modified libraries. This bias was more pronounced in N-terminal modification with AOB, compared to Cys-mediated cyclization with bDCO. (**g**–**i**) Visualization of the normalized ratio of “captured” populations compared to the population before modification. (**h**) The shape of distribution produced by comparing unmodified and bDCO-modified populations was similar to the ratio of sequences randomly sampled ten times from a sum of populations described in (**d**), (**e**) and (**f**) (red diamonds).

Within the same analysis framework, one can also efficiently analyze chemically-modified peptide libraries diversified using N-terminal ligation^47^ or chemical cyclization^11,12^. To this end, we chemically modified NT-TriNuc libraries using (i) aminooxy biotin (AOB) ligation to aldehyde generated by oxidation of N-terminal Ser and (ii) S_N_2 reaction between biotin dichloro-oxime (bDCO) cross-linker and thiols (Fig. 7a–c). After modification, we captured the entire library using streptavidin agarose beads, rinsed the beads and subjected them to PCR amplification. The NT-TriNuc library was modified in 50% and 80% yield, respectively, using AOB and bDCO. The yields were determined by a biotin capture method reported previously^11,47^. Similarly to previous reports, we used a WT phage to confirm that capturing occurs predominantly due to presence of biotin, whereas the unmodified library (or WT) was not captured in these conditions. Analysis of the library composition before modification and after chemical modification using a 20:20 plot uncovered an interesting lack of Phe-containing and Ser-containing peptides in both AOB- and bDCO-modified libraries (Fig. 7d–f). These Phe/Ser-rich peptides were present in the library before modification, but were deleted from captured populations. Analysis of the normalized ratio of the “captured” populations to the population before modification indicated that the bias against Phe/Ser was to a lower degree in thiol alkylation by bDCO and more pronounced in N-terminal modification by AOB (Fig. 7g–i). The shape of the distribution produced by comparing unmodified and bDCO-modified populations was similar to that obtained by random sampling (Fig. 7h, red diamonds).

The mechanism behind the Phe/Ser-bias is not clear, and will be investigated in our subsequent reports. We note that modification at N-terminal serine can fail for two reasons: (i) the residue is cleaved; (ii) Ser residue is not N-terminal (i.e., leader sequence or its fragment has not been cleaved in this sequence PFYSHS|SX_1_CX_2_X_3_X_4 _C). If Ser is not an N-terminal residue, it cannot be oxidized by NaIO_4_; it can neither be modified by AOB, nor captured. On the other hand, even if the leader sequence has not been cleaved, Cys is still accessible for bDCO modification. The peptide is unreactive toward bDCO only when the complete cleavage of the SX_1_CX_2_X_3_X_4 _C sequence takes place. Modification at both residues can also be sensitive to the local chemical environment but significant differences in biases between two chemically-similar residues (Ser vs. Thr) and presence of Ser in the vicinity of leader peptidase cleavage site PFYSHS|SX_1_CX_2_X_3_X_4 _C suggests that the origin of bias might stem from improper processing of the leader sequence rather than differences in chemical environment. It is possible that Ser and Phe in X_1_ or X_2_ positions lead to improper cleavage of leader peptides, frequent mis-processing of the N-terminal cleavage or cleavage of the entire displayed peptide.

## Discussion

The quality of the composition of naïve libraries is often judged by the ability to give rise to functional ligands selected from these libraries. Such indirect assessment could overlook the fundamental biases in sequence composition exhibited by the platform. Popular display platforms, such as yeast^54^, bacteria^55^, filamentous phage and phagemid^15^, T4 phage^56^, mRNA display^57^ or even different variants of the M13 phage display platform, such as pIII-Sec.^16^ vs. pIII-SRP driven display^18^ exhibit non-overlapping biases in sequence composition. Analysis of similar compact libraries in those contexts can elucidate new strategies for combining the display platforms to overcome the bias altogether. In this manuscript, analysis of the compact library made *via* trinucleotide cassette synthesis uncovered a specific loss in diversity both in transition from {**S**} to {**L**} and from {**L**} to {**N**} populations. Cloning of similar libraries into different constructs highlighted that the nature of the sequences censored in {**N**} depends on the cloning location. Libraries cloned into the N-terminus of the N1 domain of pIII censored Arg/Lys rich sequences, while libraries expressed between domains N1 and N2 of pIII protein rescued the Arg-bias. Unfortunately, a newly acquired bias towards Gly/Ser-rich sequences makes this location a less preferred site for cloning of diverse libraries. The first library-wide comparison of chemically modified and unmodified populations further uncovered biases that occur following cPTM; these biases, however, are unlikely to be the direct result of chemical modification. Rather, our analysis highlighted the possible processing defects in a specific subset of peptides displayed on the N-terminus of pIII. We hypothesize that such processing defects, such as incorrect cleavage of the signal sequence, hinder cPTM that rely on the presence of properly processed N-terminal Ser residue.

We hope that this report serves as the first step in both the open reporting of library composition, as well as the development of a standardized framework for evaluating and sharing data that describes the diversity of libraries. The characterization procedure used in this report describes a simple analysis pipeline that can be applied to many types of libraries. We propose that the cloning of a compact, complete library of the same composition into different phage constructs or other display constructs could be adopted as a standard operating procedure for characterization of the advantages and disadvantages of specific display platforms. It can also be used to test and validate new methods for overcoming these biases: for example, we demonstrated that growth-enhanced “parasite clones” can be suppressed through emulsion amplification^22,58^. Similarly, in the context of SELEX, it has been demonstrated that similar emulsion-based compartmentalization could be used to suppress “parasite” oligonucleotide sequences^27,59^. Evaluation of performance of emulsion amplification and similar methods in compact libraries of complete diversity can further confirm the ability of these methods to mitigate bias.

The analysis presented here was empowered by modern deep sequencing capabilities; still, it highlights a few of the remaining deficiencies, such as the low confidence in the accuracy of analysis of low-copy-number sets in naïve libraries. Illumina introduced new, improved chemistry for paired-end sequencing at the time of preparation of this publication. We found that the new reagents led to a significant improvement in the accuracy of sequencing. We envision that further improvements in sequencing protocols and the development of PCR-free sequencing pipelines will further boost the quality of the analysis of naïve libraries.

While short, tetrapeptide libraries are rarely used for ligand discovery, the maximum diversity of such libraries (10^5^–10^6^) is easily accessible to routine Illumina sequencing and data analysis, which makes them model libraries for investigation of library composition. The analysis of diversity of a complete, medium-size library can be particularly valuable in the characterization of new display strategies, exploration of new cloning locations (e.g., pVI, pXI of M13), the invention of dual display platforms^60^, and the expansion of display concepts into unnatural amino acids^61^ and post-translationally-modified peptides such as bicyclic peptides^10^, glycopeptides^11,12^, peptides with encoded unnatural pharmacophores^51^ and ribosomally synthesized and post-translationally modified peptides (RiPPs)^62^. Analysis of biases will in turn lead to more efficient strategies for the exploration of the sequence space using multiple display platforms of complimentary composition. Such analysis can be used in synergy with other bioinformatics tools that improve ligand discovery through retrospective comparison of identified ligands with previously discovered ligands^63,64^.

In conclusion, analysis of the NT-TriNuc, NT-SX4 and ID-SX4 libraries made via trinucleotide cassette synthesis and NNK-randomized codon uncover a minor change in diversity in transition from {**S**} to {**L**} and a strong bias in transition from {**L**} to {**N**} libraries. The nature of the censored sequences depends on the cloning location. Libraries cloned into the N-terminus of the N1 domain of pIII censored Arg/Lys rich sequences, while libraries expressed between domains N1 and N2 of pIII protein alleviated the Arg-bias but introduced new bias towards Gly/Ser-rich sequences. We are employing the library characterization pipeline reported in this study to further methods for correction of compositional biases to improve the quality of the library and to increase the reproducibility of discovery of ligands from these libraries.

## Materials and Methods

### Library design and construction

The NT-TriNuc library used in this study contains only 19 specific codons (“allowed” codons, cysteine excluded) and do not contain the other 45 codons (FC). The structure of the library is Ser-X-Cys-XXX-Cys with the X representing one of the 19 allowed codons. This library has a maximum peptide and nucleotide diversity of 19^4^ = 1.3 × 10^5^. The NT-TriNuc library makes it convenient to test the accuracy of deep sequencing results because mutations of the limited codons used in NT-TriNuc libraries results in formation of FC that should not be present in the library by design (i.e., by synthesis).

NNK-randomization is another commonly used strategy to achieve a “complete” random diversity in peptide library design. Therefore, we also generated NNK libraries. We built an N-terminally displayed library with the sequence Ser-XXXX, termed NT-SX4^44^. Previously, we hypothesized that cloning libraries within a different location on pIII could bypass the biases associated with decreased efficiency of periplasmic export of charged sequences^45^. To test this hypothesis, we also cloned a library into an intra-domain phage-display (ID-PhD) vector, between domains N1 and N2 of pIII protein^45^, termed ID-SX4.

The inserts for the NT-TriNuc libraries were constructed using ssDNA oligonucleotides purchased from TriLink BioTechnologies (San Diego, CA, USA). All other primers were purchased from Integrated DNA Technologies (Coralville, IA, USA). DNA primer 5′- CAT GGC GCC CGG CCG AAC CTC CAC C-3′ was annealed with the synthetic ssDNA library template 5′- CCC GGG TAC CTT TCT ATT CTC ACT CTT CT-X-TGT-XXX-TGT GGT GGA GGT TCG GCC GGG CGC-3′, where the X represents one of 19 allowed codons (cysteine was excluded).

The NT-SX4 libraries were prepared as described in^65^. Library ssDNA template 5′- GCG CCC GGC CGA TCC TCC TCC MNN MNN MNN MNN ACT AGA GTG GAG AAT AGA AAG GTA CCC GGG-3′ was annealed with primer 5′- CAT GCC CGG GTA CCT TTC TAT TCT C-3′. The annealed primer was extended using DNA polymerase I Klenow fragment and digested using Kpn1 and Eag1 restriction enzymes (New England Biolabs, Ipswitch, MA, USA). Purified insert DNA was ligated into linearized M13KE dsDNA vector, also digested with Kpn1 and Eag1. Ligated DNA was then transformed into electrocompetant *E.coli* TG1 (Lucigen, Middleton, WI, USA) and incubated at 37 °C for 4.5 hours with shaking. The ID-SX4 library was cloned as previously described^45^. For all subsequent titering, *E. coli* ER2738 was used (New England Biolabs, Ipswitch, MA, USA). Both TG1 and  ER2738 are amber suppressor strains that translate TAG codon as Gln.

### Chemical modification of libraries

Chemical modification of libraries using N-terminal oxime ligation^47^ and dichloro-oxime-mediated cyclization^11^ has been developed and optimized in our previous publications. To investigate possible biases in these chemical modification in the context of the entire library, we chemically modified NT-TriNuc libraries (~10^11^ pfu) using AOB and bDCO cross-linker, respectively. Reaction yields were determined by streptavidin capture of a small fraction (~10^5^ pfu) of the library and were found to be ~40–80%. After modification, the entire modified library was dialyzed exhaustively against PBS (10,000 MWCO) for 24 hours and captured using streptavidin agarose beads (Pierce, #20349). The efficiency of capture remained at ~40–80% after dialysis, indicating that the phage-biotin linkage was not hydrolyzed during dialysis. Beads that contained captured phage were washed thoroughly. Then 30 μL of 10 mM Tris-HCl and 30 μL of hexane were added to the washed beads and the mixture was shaken on a shaker (IKA, 3319000) at 3000 rpm for 10 min to extract ssDNA. Hexane was then evaporated at 70 °C on a heat block for 10 min. The released ssDNA was then separated from the beads using a pipette, then subjected to PCR amplification and sequencing as described below.

### Illumina sequencing

The steps for deep sequencing of phage libraries were similar to those described in our previous reports^22,46^. DNA originating either from synthetic oligonucleotide (Fig. 1b) or naïve/selected libraries were converted to Illumina-compatible dsDNA by PCR amplification using primers that contain built-in Illumina adapters and sequencing barcodes (Fig. 1c,d and Figure S11a–c). A 50 μL PCR reaction was set up according to manufacturer protocol (NEB, M0530) using the extracted ssDNA. The variable regions from each library were amplified and tagged with Illumina adaptor primers using 32 cycles of PCR amplification. The dsDNA PCR fragment corresponding to the expected size was confirmed and quantified using agarose gel electrophoresis. dsDNA amplicons emanating from multiple experiments were PCR-amplified with primers that contain different barcodes, mixed together, sequenced together and then distinguished using sequencing barcodes. A total of 20 ng of PCR fragments pooled from multiple experiments were purified by E-Gel (Thermo Fisher Scientific, Waltham, MA, USA). DNA was hybridized to an Illumina chip, bridge amplified and then sequenced using Illumina technology. We used both the NextSeq® 500 Mid Output Kit (150 cycles) (cat FC-404-1001) and the NextSeq® 500/550 Mid Output Kit v2 (150 cycles) (cat FC-404-2001) to sequence the reads in both the forward (F) and reverse (R) directions. FASTQ files were processed using paired-end analysis in MATLAB (See MatLab.ZIP in Supplementary Information). Raw FASTQ files (>10 Gb of data) are not included in this manuscript, but are available on request.

### Analysis of Illumina data

Supplementary information (SI) contains a detailed description of this pipeline.  All MATLAB codes (available in MatLab.ZIP in SI) with model FASTQ files are available at http://www.chem.ualberta.ca/~derda/trinucpaper/rawfiles/data/. Briefly, the pipeline starts from alignment of sequences from F and R FASTQ files, identification of barcode sequences that correspond to individual experiments and separation of aligned reads to individual files that contain sequences that map to specific sequencing barcodes. Within these files, we aligned the F and R strands and identified FA and RA sequences, allowing no more than one point mutation per adapter region. FA and RA sequences were trimmed, leaving behind library regions flanked by FA/RA. Library regions that contained identical nucleotide sequences were combined, counted and translated into peptide sequences. The final output of the pipeline is plain-text-based lists of peptide sequences and their abundances. Intermediate data files that contain F and R aligned sequences with untrimmed adapters were used to evaluate the fidelity of sequencing and processing. The newly-developed paired-end processing software supersedes our standard single-end published routine^22,46^ (Table S2 compares our single-end and paired-end processing pipelines). It allows for analysis of longer sequences and for additional quality control: a significant fraction of point-mutants emanating from a single-end pipeline can be resolved as FR-mismatches.

### Differential enrichment analysis

In the differential enrichment analysis, three factors were considered: (i) appropriate modeling of the counts using a negative binomial model; (ii) Benjamini– Hochberg (BH) correction to control the false discovery rate (FDR) at α = 0.05^52^; (iii) normalization of data across multiple replicates using the Trimmed Mean of M-values (TMM) normalization^66^. The differential enrichment analysis was performed using Bioconductor package edgeR^67,68^ (see R.zip file in the Supplementary Information for *.RMD R-code).

### Data availability

Data generated or analyzed during this study are included in this article (and its Supplementary Information files). Raw FASTQ files (>10 Gb of data) are not included in this manuscript, but are available on request.

## Electronic supplementary material

Supplementary Information

Supplementary Information

Supplementary Information

Supplementary Information
