# Supplementary material for: Compositional Bias in Naïve and Chemically-modified Phage-Displayed Libraries uncovered by Paired-end Deep Sequencing
Source: Sci Rep. 2018 Jan 19;8:1214. doi: 10.1038/s41598-018-19439-2 (PMC5775325; doi:10.1038/s41598-018-19439-2)
Supplement: Supplementary file 1 — Supplementary Information [file 41598_2018_19439_MOESM1_ESM.pdf]

Supplementary Information

Compositional Bias in Naïve and Chemically-modified Phage-Displayed Libraries uncovered by Paired-end Deep Sequencing

Bifang He[1,2,#], Katrina F. Tjhung[1,#,§], Nicholas J Bennett[1], Ying Chou[1], Andrea Rau[3], Jian Huang[2,4], Ratmir Derda[1,*]

[1] Department of Chemistry and Alberta Glycomics Centre, University of Alberta, Edmonton, AB T6G 2G2, Canada

[2] Key Laboratory for NeuroInformation of Ministry of Education, School of Life Science and Technology, University of Electronic Science and Technology of China, Chengdu 610054, China

[3] GABI, INRA, AgroParisTech, Université Paris-Saclay, 78350 Jouy-en-Josas, France

[4] Center for Information in Biology, University of Electronic Science and Technology of China, Chengdu 610054, China

[#] these authors contributed equally to this publication.

[§] present address: The Scripps Research Institute, 10550 N. Torrey Pines Rd., La Jolla, CA 92037, USA and The Salk Institute, 10010 N. Torrey Pines Rd. La Jolla, CA 92037 USA

[*] Correspondence and requests for materials should be addressed to R.D. (Email: ratmir@ualberta.ca, Tel: +1 780 492 8370)

Table of Contents

Table S1 Characteristics of sequencing data with Primer ID... S3

Table S2 Comparison of single- and paired-end processing pipeline…………………...S4

Figure S1 20:20 plot of PCR-amplified NT-TriNuc library……………........................S5

Supplementary Section S1 Description of Primer ID technology……………………...S6

Figure S2 Overview of Primer ID………………………………...................................S7

Identification and analysis of mismatches between F and R sequencing data……...S8

Evaluation of FR-mismatches as a function of sequencing chemistry………………S8

Evaluation of FR-mismatches and their relation to Phred score……………………S9

Figure S3 Description of reads in the synthetic NT-TriNuc library…………………...S10

Figure S4 Evaluation of mismatches between F/R sequencing…….........................S11

Figure S5 FR-mismatches in the NT-TriNuc libraries…………………………………S12

Figure S6 FR-mismatches in the NNK libraries S13

Figure S7 FR-mismatches in TriNuc libraries of different length S14

Figure S8 FR-mismatches in the NNK libraries S15

Figure S9 FR-mismatches in V1 kit vs. V2 kit S16

Figure S10 Phred scores and FR-mismatches S17

Figure S11 Primers for synthetic library DNA…………………………………...S18

Figure S12 Positional abundances of TriNuc codons in libraries S19

Figure S13 Description of sequence classification……...………….........................S20

Figure S14 Comparison of {**S**}, {**L**} and {**N**} of NT-SX4 libraries...............................S21

Figure S15 Comparison of NT-TriNuc and NT-SX4 libraries S22

Supplementary Section S2 Description of the paired-end processing algorithm S23

Description of all MATLAB scripts……………………………………………….S26

Table S3 Main and auxiliary MATLAB scripts……….………………………………S26

Table S4 Files used to generate images in specific figures…………………………….S31

Figure S16 Full description of Figure 5 .... …….S34

Table S1 Characteristics of sequencing data with Primer ID

Sample	No. of reads (SXCX3C)	No. of reads with "forbidden" codons	No. of reads corrected by Primer ID
Replicate 1	91,216	3327	2
Replicate 2	142,841	5028	1
Replicate 3	146,258	5207	1
Replicate 4	147,277	5359	1
Replicate 5	126,909	5007	4
Replicate 6	141,204	5677	2

Table S2 Comparison of previously published single-end and new paired-end processing pipeline

**Single-end processing pipeline**	**Paired-end processing pipeline**
Script: *rawseq.m*<br>1. Extract 500,000 lines form FASTQ file<br>2. Use forward and reverse adapter to map the variable region and barcodes.<br>3. Number the barcodes<br>Output: out0000n.txt (200 files)	Script: *fastq2aligned.m*<br>1. Extract 500,000 lines from FASTQ forward and reverse files<br>2. Map forward and reverse barcode<br>3. Concatenate the forward and reverse read and place in the AllRAWFiles/date-RxFy.txt file (sequencing date)
Script: *matchbarcode.m*<br>1. Read the out000n.txt files and distribute sequences that correspond to each barcode to individual files.<br>2. Calculate frequency of each sequence<br>3. Use name supplied in Excel file to name the output file.<br>Output: PAR/FxRy-name.txt	Script:*aligned2unique.m*<br>1. Load AllRAWFiles/date-RxFy.txt<br>2. Find vector of registry shifts for optimal alignment of each sequence, map sequences without FR-mismatches after the optimal alignment.<br>3. Save sequence mapped to AllParsedFiles/parsed_date-RxFy-name.txt (name provided in the Excel file)<br>4. Group, count and translate the sequences<br>5. Save in AllUniqueFiles/date-RxFy-name.txt
Script: *PeptideTranslator.m*<br>1. Open PAR/FxRy-name.txt, translate and trim the sequence if necessary<br>2. Save in LIB/FxRy-name.txt	

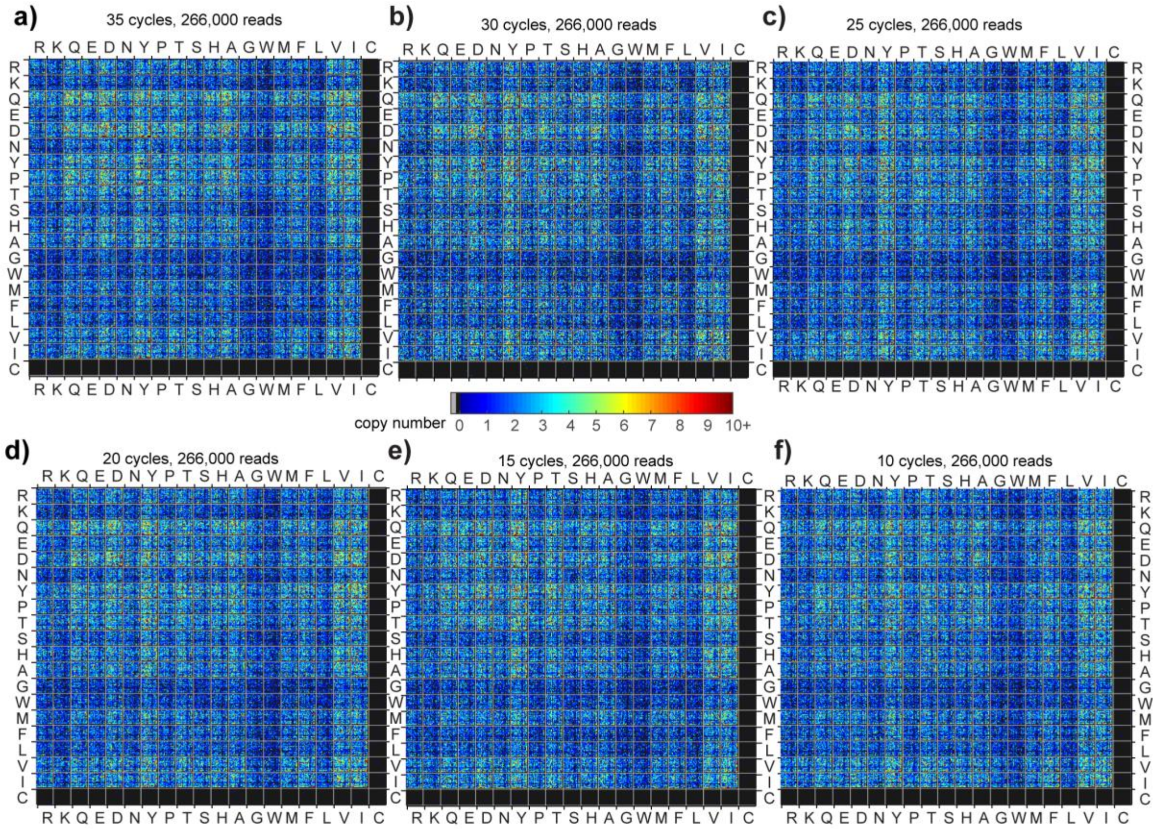

Figure S1. Distribution of individual sequences of NT-TriNuc library at different PCR cycle numbers. No major changes for individual sequences were observed when number of PCR cycles was changed from 10 to 35.

Description of Primer ID technology used in the synthetic NT-TriNuc library. We performed single-round extension of each individual DNA molecule with a primer that has a distinct Primer ID barcode (Figure S2a) composed of 8 randomized nucleotides, producing a total of 65,536 distinct combinations (4^8). If subsequent PCR produces point mutations, the errors caused by PCR could be detected as a presence of point mutants with the same Primer ID barcode. As the NT-TriNuc library only contains 19 TriNuc codons, forbidden codons caused by point mutations can be tracked and corrected by Primer ID technology.

To correct reads with forbidden codons, we first identified reads with forbidden codons, reads with allowed codons and their corresponding Primer ID barcodes. For each sequence with FC, we found sequence(s) from the allowed set that contains the same PID and calculated Hamming distance between the allowed and forbidden sequences. If two sequences were within h=1, we recorded these sequences as "corrected" (Figure S2b). Example of the reads and their codons are below:

```
Read         primerID
TTCGCTCATCAA GTTCTTCC read with forbidden codon
TTCGCTCATAAA GTTCTTCC read that has the same primer ID and no forbidden codon
************************************************************
CATGATTTCCGT CGTTATCG read with forbidden codon
CATGTTTTCCGT CGTTATCG read that has the same primer ID and no forbidden codon
************************************************************
ATGCACGACTAC CCTCTTTC read with forbidden codon
ATGCATGACTAC CCTCTTTC read that has the same primer ID and no forbidden codon
```

As shown in Table S1, only a few reads with forbidden codons were corrected by Primer ID technology (<0.1%).

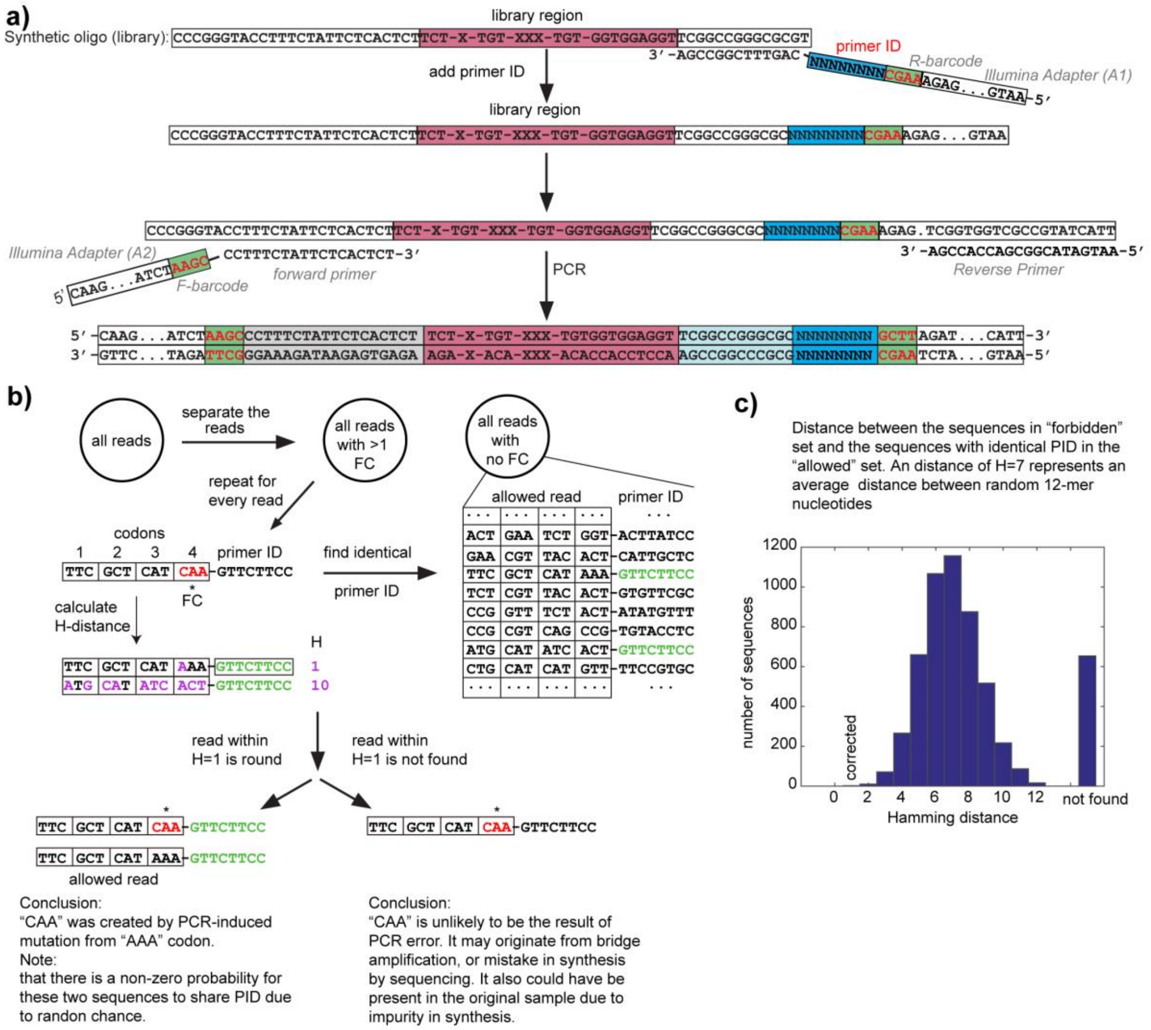

Figure S2. Overview of Primer ID. (**a**) Addition of primer IDs to synthetic oligo and PCR amplification. (**b**) Workflow of correcting reads with FC based on primer ID. (**c**) Hamming distance between the sequences in forbidden codons and the sequences with the same primer ID in the allowed sets.

Identification and analysis of mismatches between F and R sequencing data. Paired-end sequencing always contains a number of reads in which forward and reverse strands differ by one or more mismatches, referred to here as "FR-mismatches" (Figure S3). The version 1 sequencing kit issued by Illumina prior to 2015 yielded up to 80% of reads with at least one FR-mismatch. Many of them occur in very specific locations of the read (Figure S3b, S3c). The number of FR-mismatches decreased significantly when we used the V2 sequencing kit and led to the disappearance of location-specific errors. FR-mismatches in the regions remote from the library, such as a constant adapter region, had no effect on the quality of the library region. For example, analysis of sequences prepared using the less optimal V1 sequencing kit showed that the number of "false codons" in sequences that contain no mismatches in adapter regions is indistinguishable from that in reads that always contained mismatches in adapter regions (Figure S3d, blue and green). On the other hand, FR-mismatches in the library region cannot be ignored because they dramatically increased the fraction of "false codons" (Figure S3d, red).

Evaluation of mismatches between F/R sequencing data as a function of sequencing chemistry. When sequencing was performed using the Illumina V1 sequencing kit, reads with FR-mismatches constituted up to 60% of the total read population and were localized to very specific positions in the read (Figure S4b, S4c). The same FR-mismatch positions were observed when different preparations of the same library were sequenced on different instruments (Figures S5-8) or when different length and structures or encoding schemes of libraries were used (Figures S7, S8). These position-specific FR-mismatches disappeared once the V1 sequencing kit was upgraded to the Illumina V2

sequencing kit (Figure S9). We noted that a low background of position-independent FR-mismatches persisted even with the upgraded kit.

Evaluation of FR-mismatches and their relation to Phred score. The Phred score of the bases involved in FR-mismatch was a poor predictor (Figure S10). Reads with perfect Phred scores at the point of the FR-mismatch were common. Instead, lower quality Phred scores appeared several nucleotides upstream or downstream of the FR-mismatch. If the FR-mismatch happens in a random library region, *a priore* it is difficult to predict which strand should be considered as the "true" strand.

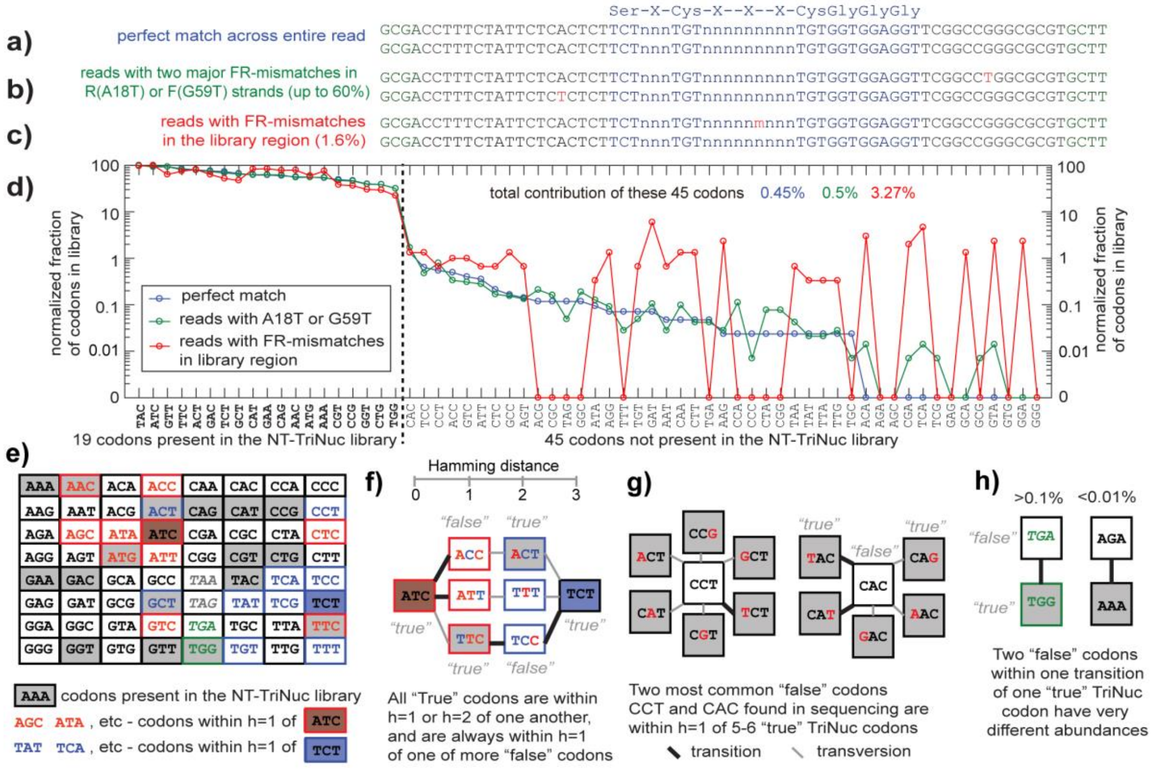

Figure S3 NT-TriNuc library with the sequence of S**X**C**XXX**C. When V1 sequencing kit was used, only ~30% of the reads were matched across the entire read in forward (F) and reverse (R) directions (**a**). (**b**) In ~70% of reads, either F or R read contained one mutation in the adapter region. (**c**) In 2% of the reads, we identified heterozygous error in library regions. (**d**) Distribution of nucleotides in synthetic NT-TriNuc library DNA. All codon counts were normalized to set the most abundant codon TAC to 100. 0.63% of the codons identified by paired-end sequencing were not present in NT-TriNuc library (aka "false"). (**e**) Codon table with TriNuc codons highlighted grey. Point mutations of ATC (red) and TCT (blue) can yield either another TriNuc codon or "forbidden" (white) codons not present in the TriNuc mix. (**f**) Hamming distance (h) plot showing that an error in TriNuc codon with h=1 can yield another TriNuc or an error located within h=1 of another "allowed" codon. For example, codon ACC can result from mutation of ATC or ACT codons. The origin of most errors, thus, cannot be traced. (**g**) Two most common erroneous codons are located within h=1 of 5 or 6 TriNuc codons. (**h**) "False" codons TGA and AGA are located within h=1 to only one "true" codon. >10-fold difference in abundance of these codons show that the number of neighbors in Hamming space is not sufficient to explain the abundance of "false" codons.

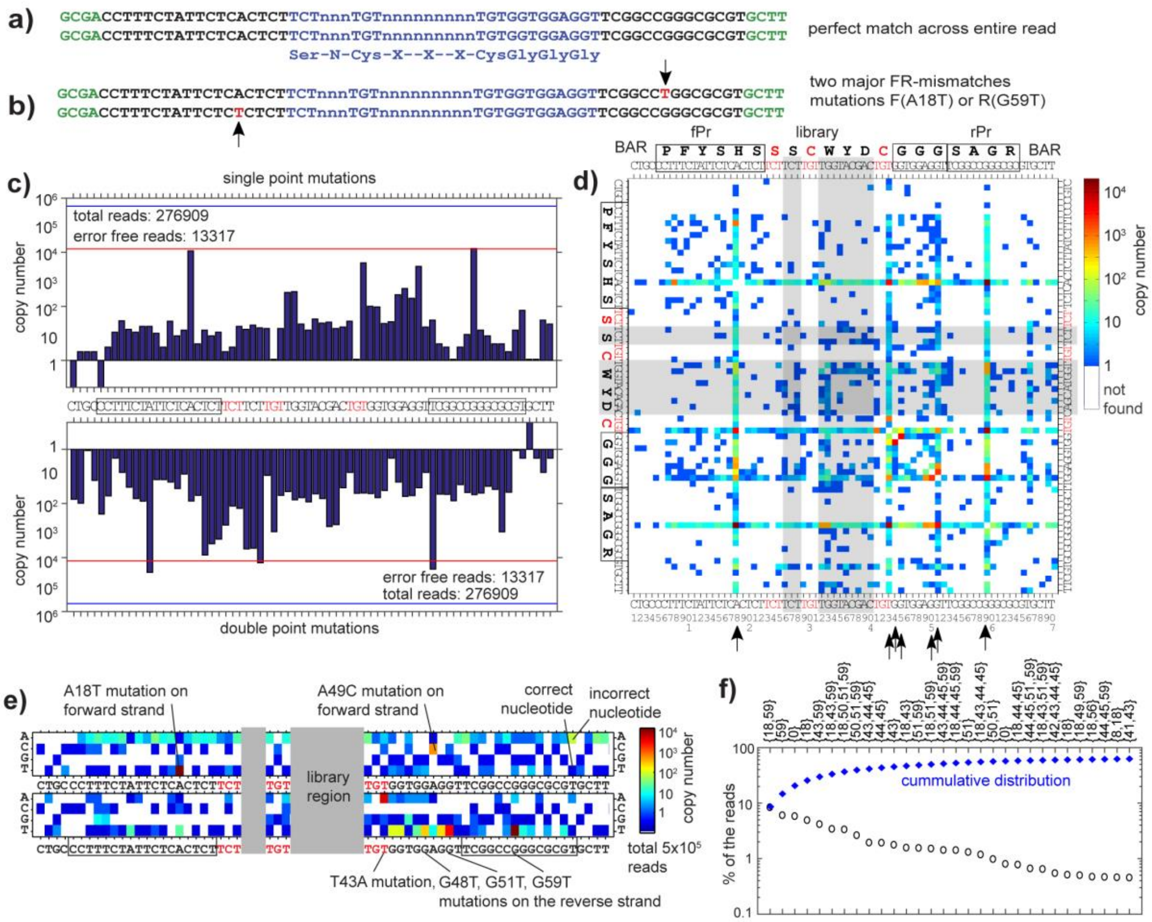

Figure S4. (**a-b**) Structure of reads with and without FR-mismatches. (**c**) Abundance of single FR-mismatches (top). In 200,000 reads, some positions have 20,000 FR-mismatches, while others have no FR-mismatches. No location is "error-free": in some locations, mismatches appear in reads that contain two FR-mismatches. (**d**) Location of double FR-mismatches. Note that most abundant errors are "benign" because they are located outside of regions of interest (grey area, library). (**e**) FR-mismatches are often specific nucleotide substitutions, often to A or T. Ignoring FR-mismatches is impossible because they constitute up to 90% of reads, yet most of them fall outside of the region of interest (in top 30 errors, which constitute 90% of all the reads, none of the errors reside in the library region, positions 26-40). All data were acquired using V1 sequencing kit.

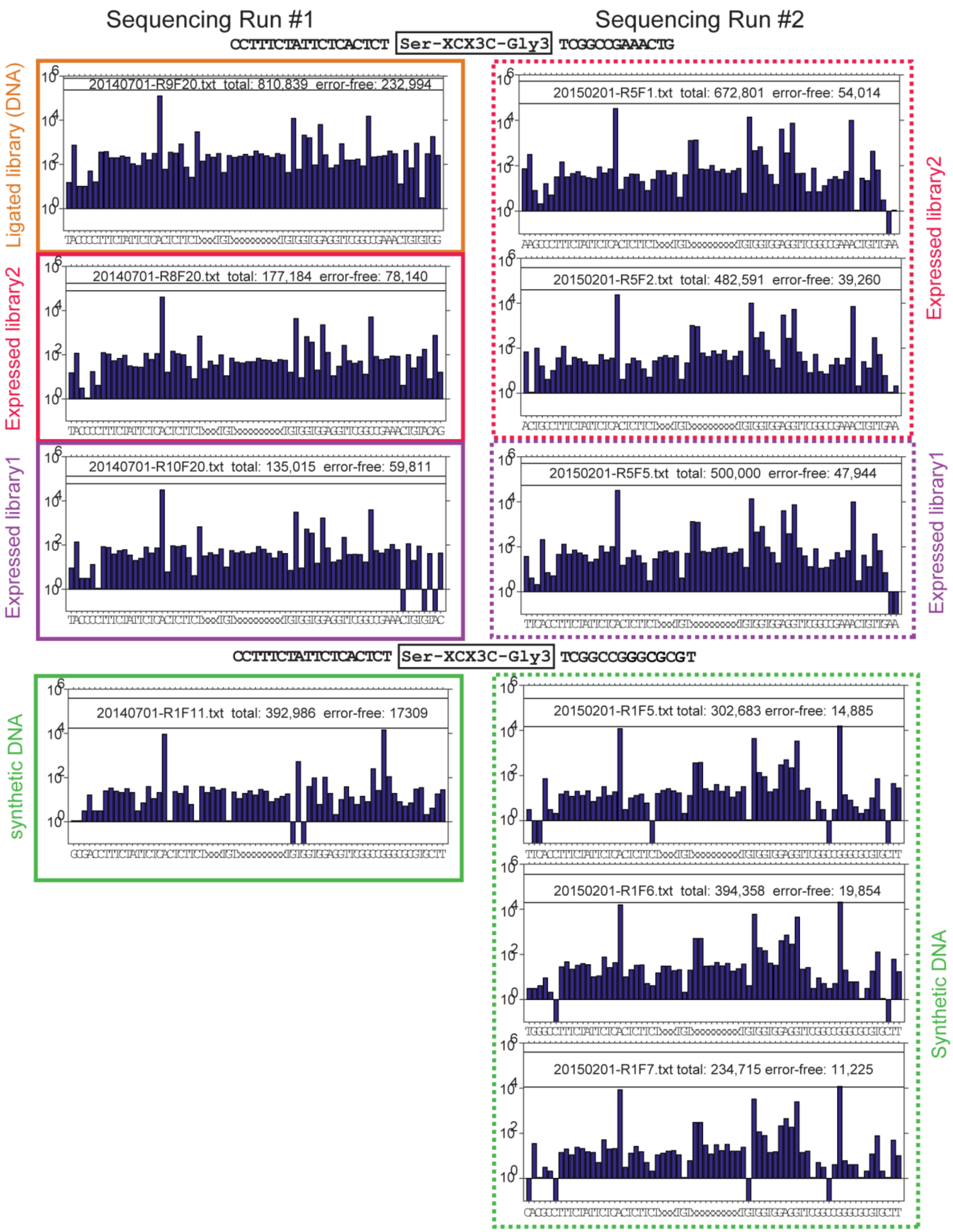

Figure S5. We observed similar FR-mismatches in the synthetic, ligated, and naïve NT-TriNuc library. FR-mismatches in adapters were similar in different sequencing runs.

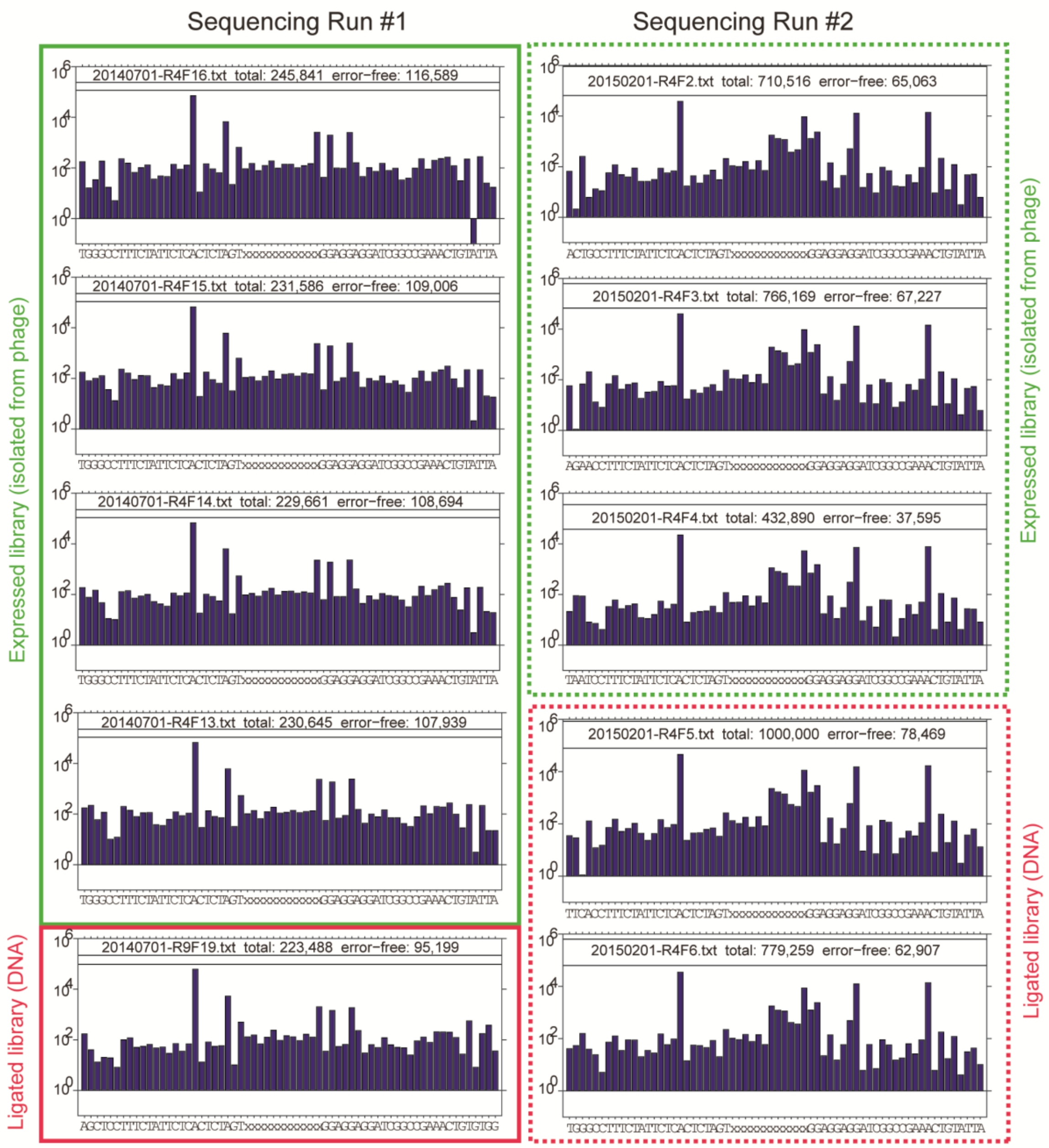

Figure S6. We observed similar FR-mismatches in the synthetic, ligated, and naïve NNK-library. FR-mismatches in adapters were similar in different sequencing runs. Errors in close proximity to the library differed from run to run.

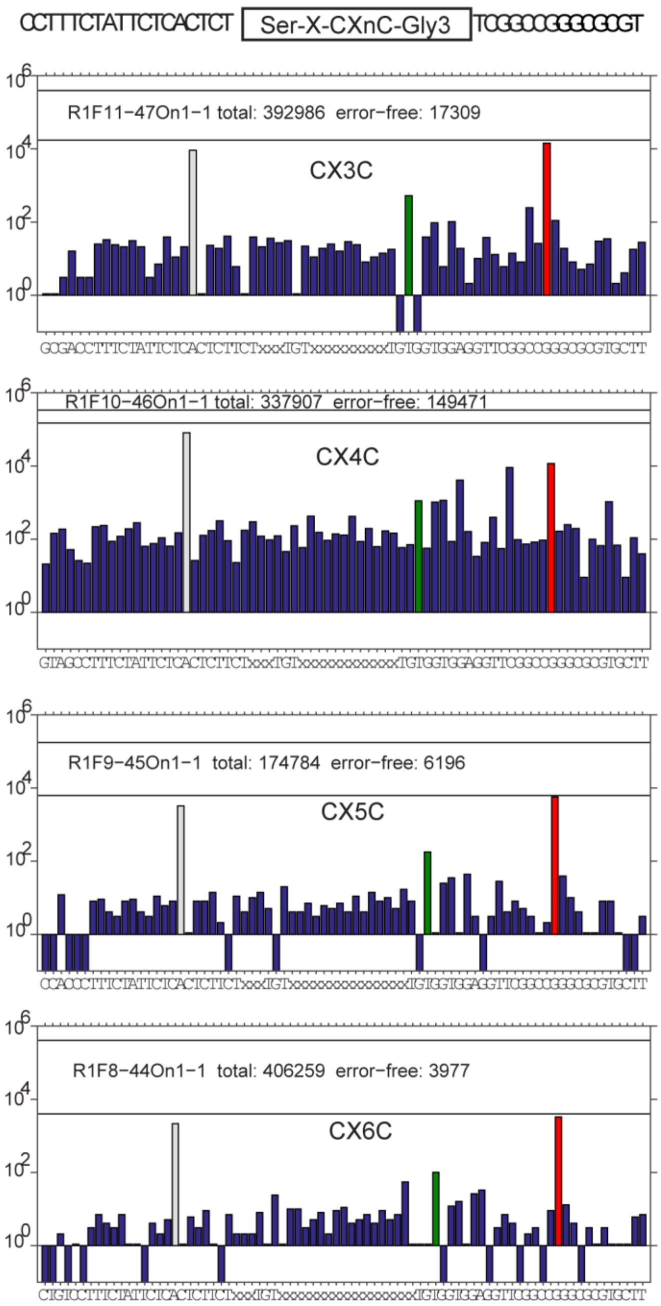

Figure S7. We observed similar FR-mismatches in libraries of different length. In all cases, major FR-mismatches were in the constant adaptor region outside of the library region and in the constant Cys.

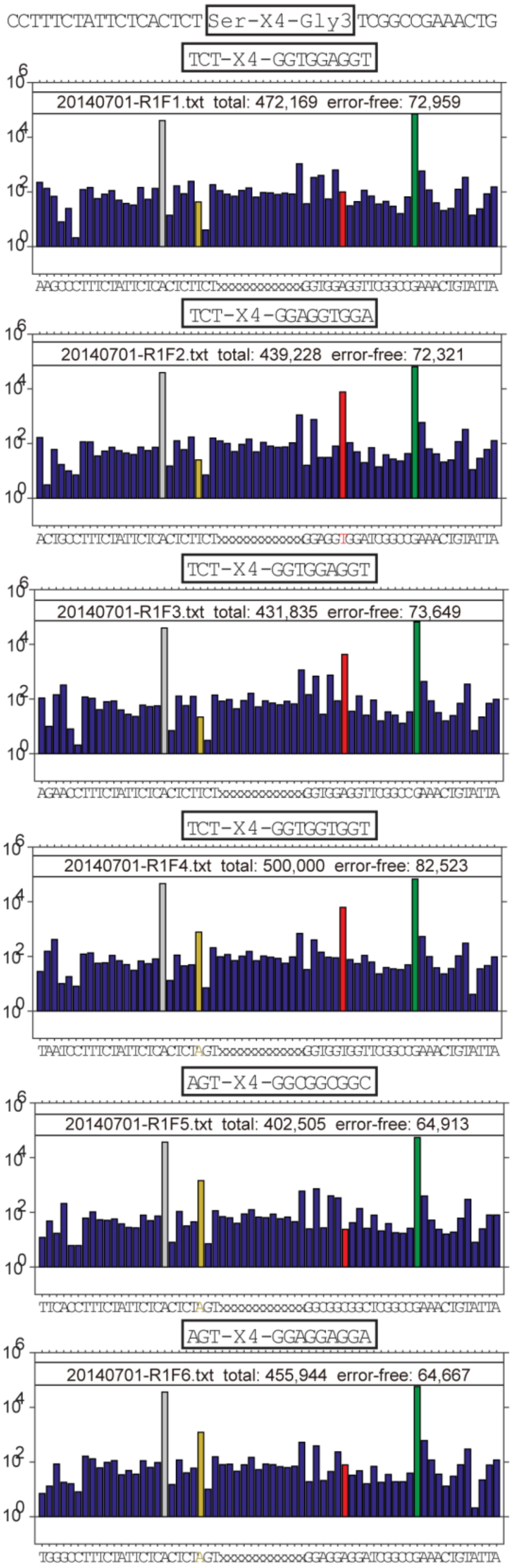

Figure S8. Mismatches in libraries with different spelling of constant Gly-Gly-Gly region in the vicinity of the library.

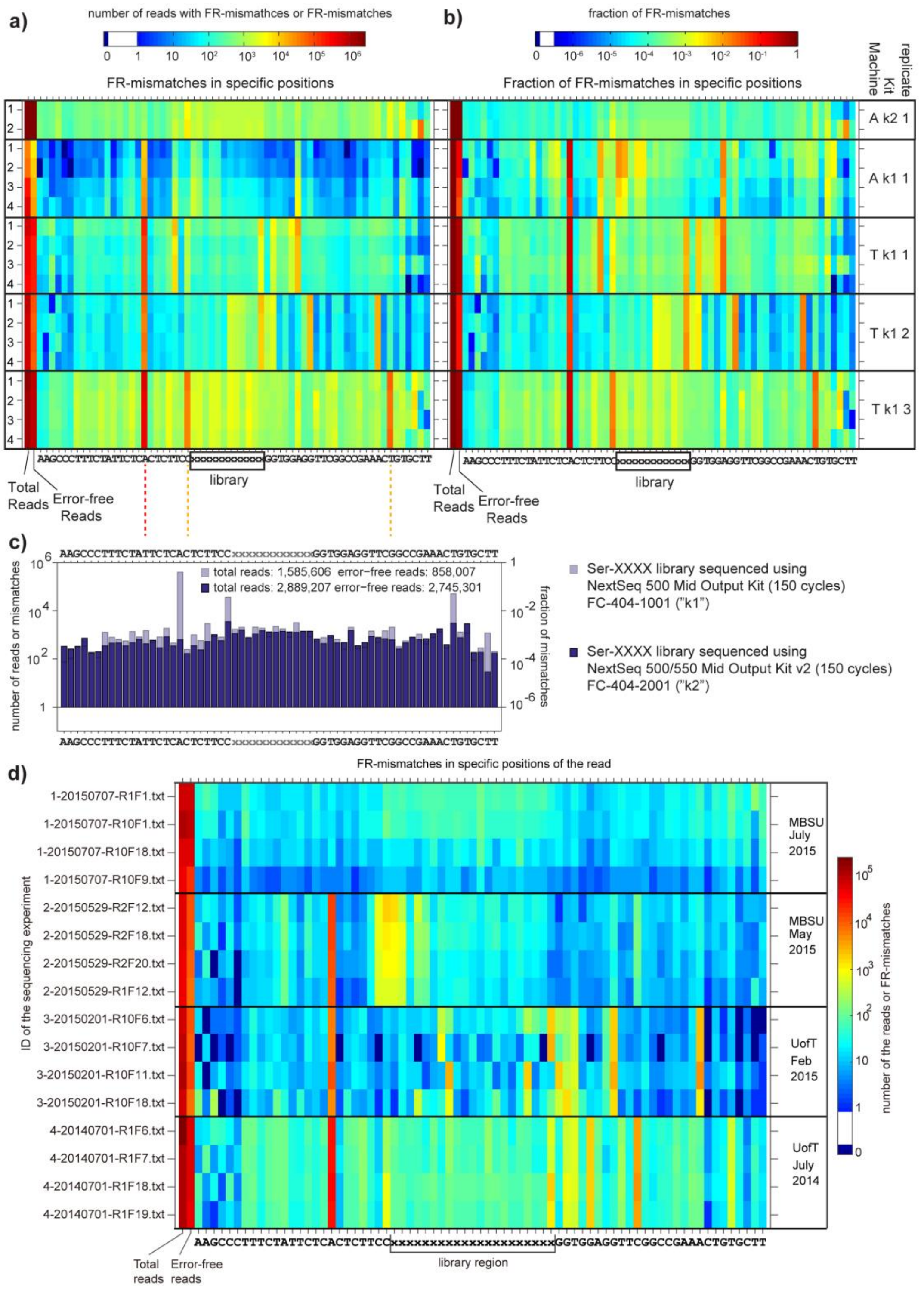

Figure S9. Mismatches decrease dramatically with introduction of a new sequencing kit in June 2015. Note that only major FR-mismatches disappear. The minor FR-mismatches across the read remain largely unchanged (**a-c**). (**d**) With V1 sequencing kit, the same FR-mismatches were observed in sequencing with different instruments (Molecular Biology Service Unit "MBSU" in Edmonton vs. Donnelly Center at the University of Toronto "UofT").

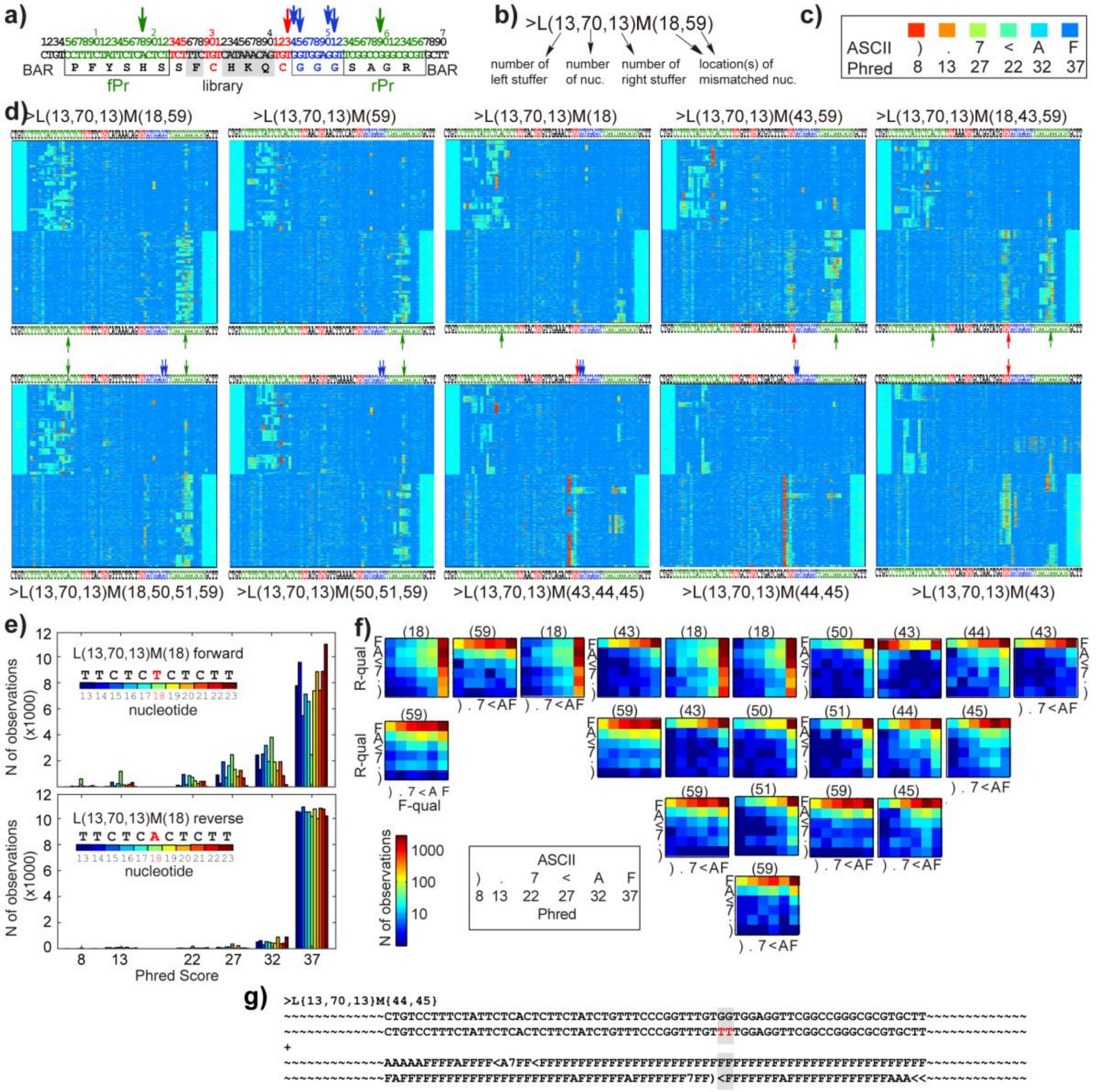

Figure S10. (**a-d**) Phred score is weakly correlated with mismatches, but it cannot be used to identify the mismatch unambiguously. Low scores appear around the mismatch, but they also appear around other areas that do not have a mismatch. (**e**) Phred score is sometimes lower at the mismatch or at the surrounding nucleotides, but in many cases it is not. In 5000 out of 150,000 observations, the score at the position of mismatch is 37, which corresponds to 99.98% base call accuracy and the highest base call accuracy observed in this sequencing run. (**f**) In some cases, both forward and reverse strands contain a perfect Phred score at the point of FR-mismatch. (**g**) An example of such a read where two FR-mismatches side-by-side exhibit perfect or nearly perfect Phred scores on both strands.

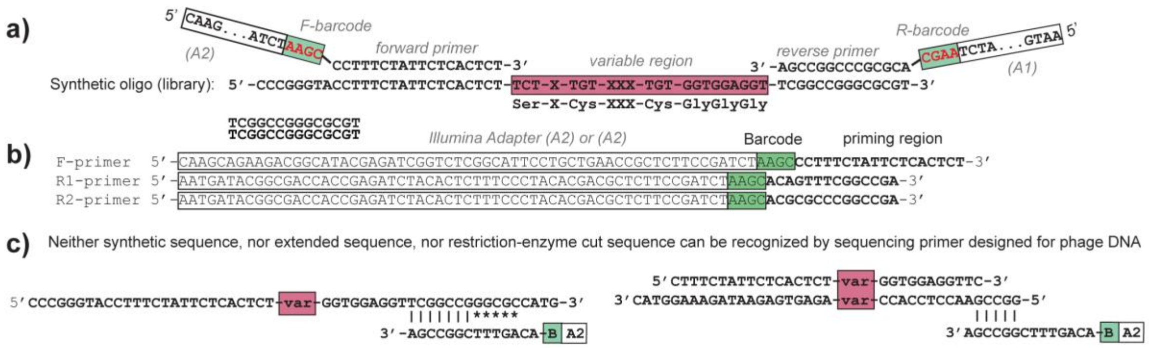

Figure S11. Primers for synthetic library DNA. (**a-c**) Design and sequence of primer that amplifies synthetic DNA. Primers that amplify ligated DNA do not recognize synthetic, cut and non-ligated DNA.

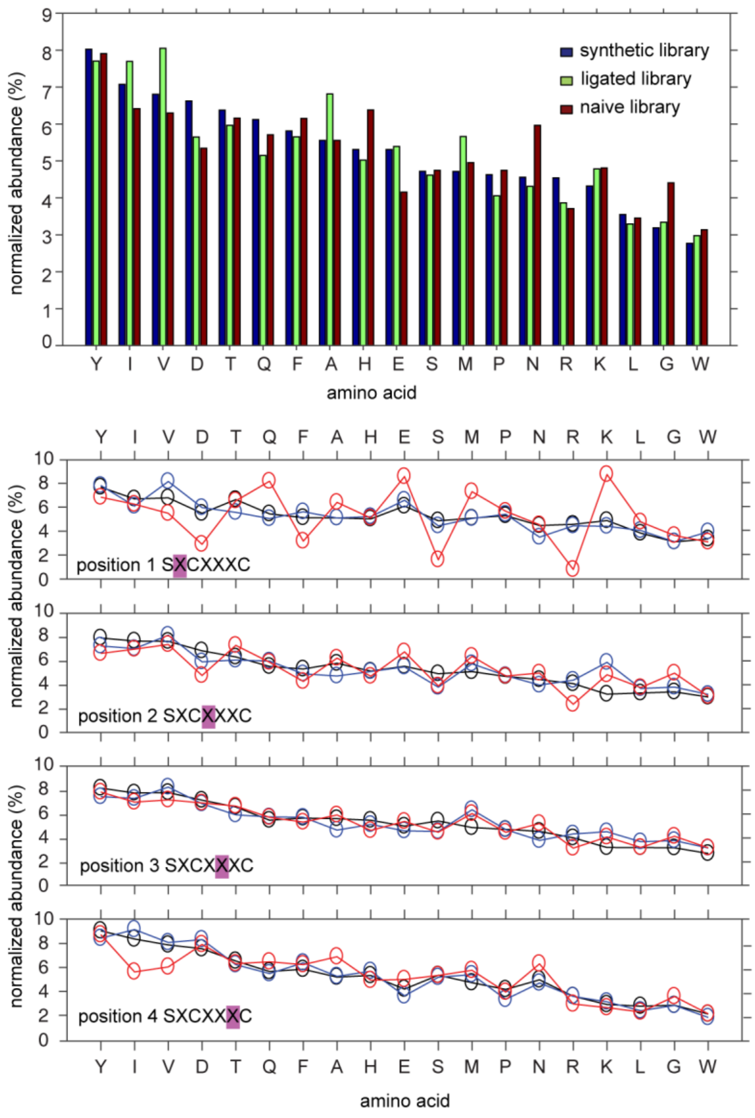

Figure S12. Positional abundances of TriNuc codons in synthetic, ligated, and expressed libraries. Top panel describes abundance of codons in all positions (copied from main text Figure4a). Panels below represent abundance of TriNuc codons in every position of the library. There is only a mild variation in the bias in the synthetic (black) and ligated libraries (blue), and much stronger positional dependence in the expressed libraries (red). For example, the bias against R-codon is the strongest at the N terminal position and it decreases towards the C-terminus.

a)

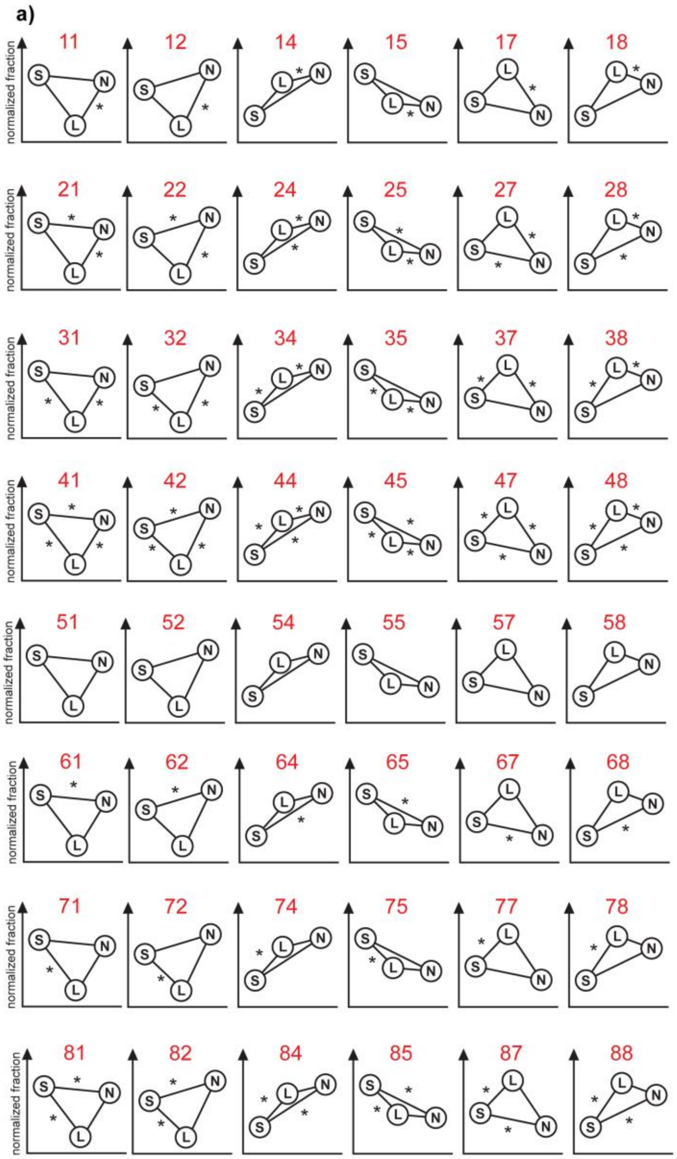

b)

Type	Seq	S1	S2	S3	S4	L1	L2	L3	L4	N1	N2	N3	N4	#
UPS55	SACFDWC	1	0	1	2	0	0	0	0	0	0	0	0	2966
UPS65	SPCGGGC	0	3	2	3	0	0	0	0	0	0	0	0	980
UPS85	SKCLDHC	1	5	4	3	0	0	0	0	0	0	0	0	214
UPL17	SFCLRRC	0	0	0	0	2	1	3	1	0	0	0	0	71
UPL37	SKCWWVC	0	0	0	0	2	3	3	3	0	0	0	0	28
UPL57	SRCMMWC	0	0	0	0	1	0	0	0	0	0	0	0	381
UPN22	SACGWDC	0	0	0	0	0	0	0	0	1	3	4	3	34
UPN52	SMCNRNC	0	0	0	0	0	0	0	0	0	0	1	0	81
UPN62	SDCWGHC	0	0	0	0	0	0	0	0	1	2	1	2	9
UAS24	SFCNHGC	0	0	0	0	0	2	2	1	3	4	7	7	39
UAS38	SFCWDRC	0	0	0	0	3	3	5	4	0	0	0	1	7
UAS54	SWCFIWC	0	0	0	0	0	1	0	0	1	1	0	1	72
UAS58	SDCWQPC	0	0	0	0	2	0	2	0	0	1	2	0	147
UAS64	SLCNPIC	0	0	0	0	2	0	1	1	4	3	2	3	108
UAS68	SGCFVWC	0	0	0	0	1	2	2	1	2	0	1	2	16
UAS78	SHCLPTC	0	0	0	0	1	3	3	1	0	1	1	2	7
UAS84	SVCQGMC	0	0	0	0	0	3	3	2	2	3	3	4	7
UAS88	SMCYPWC	0	0	0	0	1	3	1	3	2	0	3	4	16
UAL11	SACDQMC	2	3	1	4	0	0	0	0	3	0	3	3	117
UAL12	SACKPDC	2	2	1	2	0	0	0	0	3	3	0	3	802
UAL22	SPCLWQC	2	1	4	1	0	0	0	0	7	9	16	16	448
UAL31	SDCVRGC	2	3	6	2	0	0	0	0	2	2	4	4	123
UAL32	SPCGSEC	3	6	3	0	0	0	0	0	2	4	6	6	89
UAL41	SPCDQYC	11	7	6	5	0	0	0	0	4	1	4	3	7
UAL42	SHCHNKC	3	3	2	3	0	0	0	0	11	12	19	20	26
UAL51	SACEESC	4	2	0	0	0	0	0	0	0	1	1	1	1447
UAL52	SKCMGGC	0	0	1	0	0	0	0	0	0	0	2	0	828
UAL61	SGCAMSC	2	5	0	3	0	0	0	0	0	0	0	1	42
UAL71	SACAWDC	1	1	3	6	0	0	0	0	0	2	1	0	90
UAL81	SGCQNFC	1	1	2	7	0	0	0	0	1	0	0	0	95
UAN17	SACAGNC	1	2	1	1	1	1	1	3	0	0	0	0	3507
UAN25	SACAAAC	2	8	1	4	0	1	2	2	0	0	0	0	4506
UAN27	SACAGFC	1	1	7	0	0	4	3	4	0	0	0	0	5618
UAN37	SECNPGC	0	0	1	0	5	1	4	2	0	0	0	0	51
UAN45	SDCAMDC	6	6	13	4	0	3	1	1	0	0	0	0	10
UAN47	SDCVVSC	5	4	5	7	10	10	8	12	0	0	0	0	31
UAN55	SACADPC	1	0	2	1	0	1	0	0	0	0	0	0	4704
UAN57	SACANRC	0	1	1	1	1	1	1	1	0	0	0	0	3954
UAN65	SACAAWC	4	2	2	0	1	0	1	0	0	0	0	0	7089
UAN85	SACEINC	4	2	9	3	1	0	0	0	0	0	0	0	83
COM11	SACANHC	4	6	3	6	0	0	2	1	6	5	4	2	103
COM12	SACADKC	2	4	7	2	3	2	0	1	7	5	5	8	2926
COM17	SACAEFC	5	3	8	3	5	5	2	8	2	1	3	1	4172
COM18	SACARPC	0	0	1	4	3	5	5	3	1	1	1	3	133
COM22	SACAAQC	5	2	3	1	1	1	1	0	3	8	8	9	4126
COM24	SACAQNC	1	1	0	2	0	3	1	0	7	9	9	12	3043
COM25	SACAATC	8	3	4	5	5	4	5	1	1	0	0	0	1883
COM27	SACAAKC	2	5	2	5	3	3	2	3	1	1	0	1	3152
COM31	SACAHYC	7	9	9	6	2	0	1	0	8	1	5	5	115
COM32	SACAPYC	6	3	11	5	1	1	1	1	4	7	8	10	77
COM37	SACESFC	3	2	5	0	5	7	5	9	2	2	3	2	162
COM38	SACDRFC	4	4	3	4	7	9	9	10	3	4	5	4	182
COM42	SACAIRC	5	4	8	5	0	1	1	0	7	10	12	11	18
COM44	SECGGFC	1	1	1	1	3	3	5	7	8	14	18	19	37
COM45	SDCDIKC	10	15	8	10	1	4	3	3	0	0	2	1	5
COM47	SACVPIC	4	7	3	2	8	8	15	9	0	1	2	0	32
COM48	SLCLAYC	1	1	2	1	7	10	14	9	6	4	4	7	8
COM51	SACAADC	4	10	9	8	1	1	3	3	8	4	1	2	9689
COM52	SACAAGC	2	1	1	3	2	0	0	0	4	2	3	2	9944
COM54	SACADVC	5	5	5	1	3	5	3	3	3	5	4	6	7445
COM55	SACAAHC	2	3	3	2	0	0	3	2	0	0	0	2	12411
COM57	SACADAC	2	0	2	3	1	2	1	2	3	1	1	1	12166
COM58	SACADWC	0	1	0	3	2	1	1	1	2	1	1	1	8220
COM61	SACADEC	1	10	6	3	0	0	2	1	4	0	1	1	311
COM62	SACGRFC	2	0	2	0	0	1	1	1	4	2	3	5	67
COM64	SACAAFC	2	4	6	5	4	5	6	3	10	6	13	14	3531
COM65	SACAAIC	7	5	9	5	3	4	3	4	4	1	0	2	6212
COM67	SACAGGC	4	2	5	0	1	2	3	3	1	0	0	1	86
COM68	SACAVSC	1	2	3	3	6	5	4	7	5	5	5	7	153
COM71	SACAVVC	6	4	14	3	2	0	2	1	2	5	3	4	103
COM78	SACESVC	4	1	3	1	7	4	9	7	5	2	5	6	125
COM81	SACAQDC	6	5	7	4	1	1	0	0	1	0	1	1	186
COM84	SACGSSC	1	1	0	1	4	4	5	6	6	4	7	8	131
COM85	SACAFKC	9	8	6	5	0	1	0	1	0	0	2	0	150
COM88	SACGEGC	0	1	0	0	2	2	3	4	3	3	3	4	155

Figure S13. Description of sequence classification. (**a**) Sequences can be clustered into 48 classes based on relations of normalized fractional abundance of this sequence in synthetic (**S**), ligated (**L**) and naïve set (**N**), and significance of that relationship. For example, sequence in class 11 is significantly enriched in **N**aïve library when compared to **L**igated (i.e., **N**>**S***, where * represents statistical significance). The abundance of this sequence in **S** is higher than in **L** or **N** but these differences are not significant (i.e., **S**>**L**$^{n.s.}$ and **S**>**N**$^{n.s.}$). Other classes, e.g., class 22, can be described similarly: **S**>**L**$^{n.s.}$, **L**<**N***, **S**>**N***, and so on. (**b**) A list of representative sequences from each class. Numbers represent the copy number of each sequence in four replicates of sequencing of synthetic library (S1-S4), ligated library (L1-L4) and naïve library (N1-N4). The last column represents the number of unique sequences in each class. Types of classes not listed in the classification were not found (e.g., COM15, COM41, etc).

We note that several of the classes are not possible (e.g., COM21, COM28, COM34, COM35, COM82, COM87) but they are included in the classification table for continuity reasons.

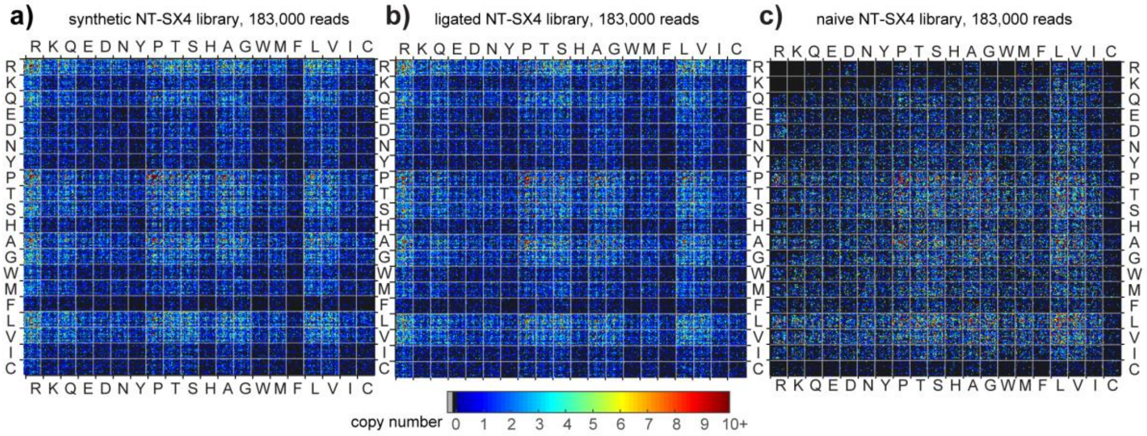

Figure S14. The 20:20 plot of a sample of 183,000 reads from deep sequencing for (**a**) synthetic, (**b**) ligated and (**c**) naïve NT-SX4 display libraries. The number of reads sampled represents 1× coverage of theoretical peptide diversity (1.6×10^5) of NT-SX4 library.

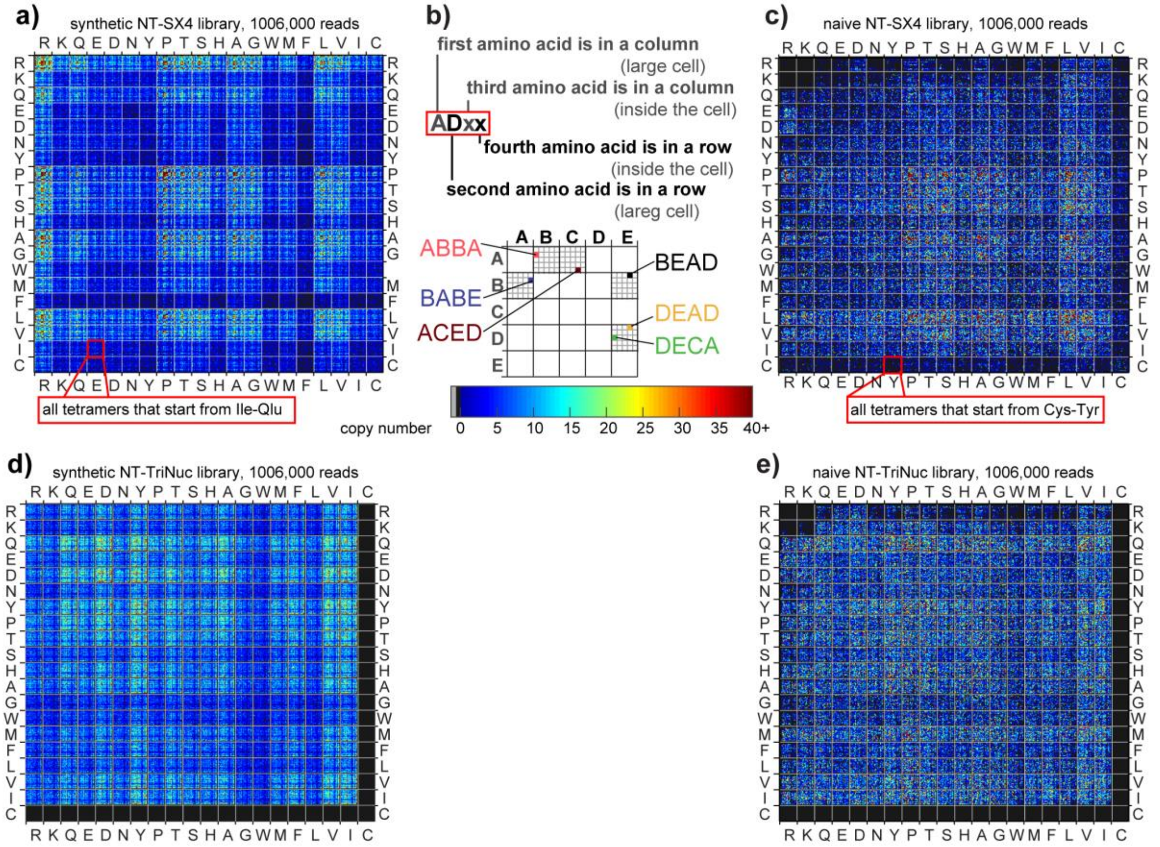

Figure S15. The 20:20 plot of a sample of 1 million reads from deep sequencing for (**a**) synthetic and (**c**) naïve NT-SX4 display libraries. We selected a sample of 1.0 million reads to represent ~6× coverage of peptide diversity (1.6×10^5) of NT-SX4 and allow comparison to 20:20 plots of NT-TriNuc libraries plotted with the same number of reads. (**d**) Synthetic and (**e**) naïve NT-TriNuc libraries. (**b**) Description of a plot showing each peptide in the library as a unique pixel in a specific location. A model 5x5 letter plot with 25x25=625 pixels describes the location of common four-letter words that contain the letters A, B, C, D and E.

Description of the paired-end processing algorithm. We first aligned forward (F) and reverse (R) reads and retained sequences that had at least 15 matches between the forward and reverse reads. Then we inspected those sequences with no FR-mismatches for the presence of F and R adapter sequences, allowing one mismatch within the adapter regions. The MATLAB code is shown below. All functions and files used in the below MATLAB script are provided as a part of Supplementary Information.

Folder: MatLab/Illumina Processing

% This folder contains all scripts and files for processing FASTQ files according to the workflow in **Figure 2**. Scripts are started by **paired_end_processing.m** script. Name(s) of FASTQ files and directories in which they are stored have to be defined in the script:
Line 27: indir = './20170504';
Line 40: rname = { 'DERDA-20170504_S1_L001_R1_001.fastq.gz' };
Line 43: fname = { 'DERDA-20170504_S1_L001_R2_001.fastq.gz' };

Once started, the script runs through the remaining 4 scripts in the same folder and 22 scripts in the **MatLab/Illumina Processing/auxiliary** folder and generates text-based status of the processing. Files will be placed in the newly created folders **AllRAWFiles/**, **AllParsedFiles/**, **AllUniqueFiles/** and **AllTableFiles/** located within the same folder as the script.

```
% This is the top-level script for processing deep-sequencing data from Illumina.
% It's function is to perform preliminary parsing of paired-end reads.

% Authors: Ratmir Derda, Bifang He. Annotated by J Maxwell Douglas and Bifang He
% Last Modifed: June 13th, 2017


clc;
clear;
addpath (genpath(pwd));

load SXY.mat;

% define the directory of all library types
AllLibraries  = './All classes of libraries';
%%%%%%%%%%%%%%%%%%%%%%%%%%%%%%%%%%%%%%%%%%%%%%%%%%%%%%%%%%%%
%%%%%%%%%%%%%%%%%%%%%%%%%%%%%%%%%%%%%%%%%%%%%%%%%%%%%%%%%%%%

%% USER SECTION

% Place the excel file and FASTQ files into the ./20170514 folder
% MATLAB then can find the excel and FASTQ files inside of the
% directory

indir = './20170504'; % directory where the excel and FASTQ.GZ files are
xlsname = dir(fullfile(indir, '20170504.xlsx'));

% This is the name of the excel sheet within the excel file.
sheetname = '2017-05-04';

% all aligned files from this run will have this prefix; change it to date
% of sequencing; do not remove the '-*', just modify the date.
files = '20170504-*.txt';

% List your fastq.gz files below for the rname and fname variables.
% Make sure to match ...R1_001.fastq.gz files to rname and
%                     ...R2_001.fastq.gz files to fname.
rname = {'DERDA-20170504_S1_L001_R1_001.fastq.gz'};
```

```
fname = {'DERDA-20170504_S1_L001_R2_001.fastq.gz'};


%%%%%%%%%%%%%%%%%%%%%% End of User defined section %%%%%%%%%%%%%%%%%%%%%%%%
%% Do not change anything below unless you know what you are
doing  %%%%%%%%%%%%%%%%%%%%%%%%

AllRAWFiles = './AllRAWFiles';
AllUniqueFiles = './AllUniqueFiles';
AllParsedFiles = './AllParsedFiles';
AllTableFiles = './AllTableFiles';


%%%%%%%%%%%%%%%%%%%%%%%%%%%%%%%%%%%%%%%%%%%%%%%%%%%%%%%%%%%%
%%%%%%%%%% Definition of sequencing barcodes; do not change %%%%%%%%%%%%%%%%%%%%%%%%%
%%%%%%%%%%%%%%%%%%%%%%%%%%%%%%%%%%%%%%%%%%%%%%%%%%%%%%%%%%%%
B.FB={'AAGC'; 'ACTG'; 'AGAA'; 'TAAT'; 'TTCA'; 'TGGG'; 'CACG'; 'CTGT'; 'CCAC'; 'GTAG';
      'GCGA'; 'GGTT'; 'AATA'; 'ATTT'; 'ATGG'; 'ACCA'; 'ACGT'; 'AGTC'; 'AGCT'; 'TACC'};

B.RB={'GCTT';'CAGT';'TTCT';'ATTA';'TGAA';'CCCA';'CGTG';'ACAG';'GTGG';'GTAC';
    'TCGC';'AACC';'TATT';'AAAT';'CCAT';'TGGT';'ACGT';'GACT';'AGCT';'GGTA'};
%%%%%%%%%%%%%%%%%%%%%%%%%%%%%%%%%%%%%%%%%%%%%%%%%%%%%%%%%%%%
%%%%%%%%%%%%%%%%%%%%%%%%%%%%%%%%%%%%%%%%%%%%%%%%%%%%%%%%%%%%

newline = [char(13) char (10)]; % enforse the Windows type newline!


% This part parses the FASTQ file, extracts raw sequences and do alignment

fastq2aligned('indir', indir, 'AllExcelFiles',indir,'fname', fname,...
              'rname', rname,'outdir',AllRAWFiles,'chunk',500000, ...
              'saveNOmatch', 0,'saveNObar', 0,'saveRAW', 0, ...
              'barcodes', B, 'XlsName', xlsname, 'SheetName',sheetname, ...
              'delimiter','@','NMismatches',4,'minimumMatch',15, 'newline', newline);

 %%%%%%%%%%%%%%%%%%%%%%%%%%%%%%%%%%%%%%%%%%%%%%%%%%%%%%%%%%%%
 %%%%%%%%%%%%%%%%%%%%%%%%%%%%%%%%%%%%%%%%%%%%%%%%%%%%%%%%%%%%


 if strcmp(files,'all')
      Nucname = dir(fullfile(AllRAWFiles,'*-R*F*.txt'));
 else
      Nucname = dir(fullfile(AllRAWFiles,files));
 end

% This function converts an excel table of sample names to the type of
% library and separates the samples based on this distinction.
[X, Samples] = name2librarytype('AllExcelFiles ',indir, ...
                                'XlsName', xlsname,...
                                'SheetName', sheetname,...
                                'sdb_numbers', SDB_ids);
valueSet =   {'SDB','MXY'};
keySet = [1, 2];
mapObj = containers.Map(keySet,valueSet);

% For each library type identified, generate UNIQUE and PARSED files.
for i=1:size(X,1)

    if X(i,1)
        % extract the name of the library from the XLS name
        NameofLibraryString = mapObj(i);

        % load the structure
        load(fullfile(AllLibraries,  NameofLibraryString ));

        %since the structures that you load have different names, you want to
        % convert them to one structure that has the same name. this
        % "MyStructure" variable can be passed as structure to the next
        % function. You cannot pass a name of the structure, you have to pass
        % the structure itself.
```

```
        eval(['MyStructure = ' NameofLibraryString ]);


        disp(['Processing ' files ' using '  NameofLibraryString ]);

        % Create parsed files with untrimmed adapters and save them to the
        % 'AllParsedFiles' folder. Then trim the adapters and agglomerate
        % counts for each unique sequence in the file and save these new
        % files to the 'AllUniqueFiles' folder.
        aligned2unique('indir', AllRAWFiles,...
                'files',files,'samplenames', Samples{i},...
                'unidir', AllUniqueFiles, 'parsedir', AllParsedFiles,...
                'library', MyStructure,...
                'Barcodes', B, 'adaptermatch', 'mutant',...
                'chunk',500000, 'newline', newline);
    end
end


%%%%%%%%%%%%%%%%%%%%%%%%%%%%%%%%%%%%%%%%%%%%%%%%%%%%%%%%%%%%%
%%%%%%%%%%%%%%%%%%%%%%%%%%%%%%%%%%%%%%%%%%%%%%%%%%%%%%%%%%%%%

% Create a table file based on two character identifier for each individual
% with samples on this sequencing run. Tables are built from the UNIQUE
% files. Each table file contains all of an individual's samples. Table
% files are saved to the 'AllTableFiles' folder.

tags2table('indir', AllUniqueFiles, 'outdir', AllTableFiles, 'files', files )

% File End
```

Description of all MATLAB scripts
Folder: MatLab
This folder contains MATLAB scripts named *MakeFigureX.m*, where X is the number of a specific figure. Each script is self-sufficient. The data used to generate figures are in the folder **MatLab/data/**. Running the script generates the image in a specific figure. All auxiliary scripts are placed in the folder **MatLab/auxiliary/**. All MATLAB scripts are available at http://www.chem.ualberta.ca/~derda/trinucpaper/ (denoted as **URL** below)

% All scripts were written by Ratmir Derda and Bifang He; any use of these scripts or portions of these scripts should be acknowledged by reference to this paper. Thank you!

Table S3 A list of all main and auxiliary MATLAB scripts

MATLAB script	Description
`MakeFigure3a.m`	This script uses NT-TriNuc_filtered_S10-35cycles.txt as input and plots panel a of Figure 3.
`MakeFigure3b.m`	This script uses Data_Figure3B.xls as input and plots panel b of Figure 3.
`MakeFigure3c.m`	This script uses NT-TriNuc_filtered_S10-35cycles.txt as input and plots panel c of Figure 3.
`MakeFigure4a.m`	This script uses NT-TriNuc_filtered_SLN.txt as input and plots panel a of Figure 4.
`MakeFigure4b.m`	This script uses NT-TriNuc_filtered_SLN.txt as input and plots panel b of Figure 4.
`MakeFigure4def.m`	This script uses NT-TriNuc_filtered_SLN.txt as input and plots panels d, e and f of Figure 4.
`MakeFigure5hij.m`	This Script uses files de_SvsL.txt, de_SvsN.txt, de_LvsN.txt, available for download from **URL** as input and plots panels h, i and j of Figure 5.
`MakeFigure6ab.m`	This script uses NT-SX4_filtered_SN.txt as input and plots panels a and b of Figure 6.
`MakeFigure6c.m`	This script uses ID-SX4_filtered_N.txt as input and plots panel c of Figure 6.
`MakeFigure7def.m`	This script uses YC_filtered.txt as input and plots panels d, e and f of Figure 7.
`MakeFigure7ghi.m`	This script uses YC_filtered.txt as input and plots panels g, h and i of Figure 7.
`MakeFigure7hsampling.m`	This script uses YC_filtered.txt as input and plots panel h (red diamonds) of Figure 7.
`MakeFigureS1abcdef.m`	This script uses NT-TriNuc_filtered_S10-35cycles.txt available for download from **URL** as input and plots panels a, b, c, d, e and f of Figure S1.
`MakeFigureS3d.m`	This script uses 20150201-R1F8.txt available for download from **URL** as input and plots panel d of Figure S3.

**MakeFigureS4c.m**	This script uses 20150201-R1F8.txt available for download from **URL** as input and plots panel c of Figure S4.
**MakeFigureS4d.m**	This script uses 20150201-R1F8.txt available for download from **URL** as input and plots panel d of Figure S4.
**MakeFigureS4e.m**	This script uses 20150201-R1F8.txt available for download from **URL** as input and plots panel e of Figure S4.
**MakeFigureS4f.m**	This script uses 20150201-R1F8.txt available for download from **URL** as input and plots panel f of Figure S4.
**MakeFigureS5.m**	This script uses 20140701-R1F11.txt, 20140701-R9F20.txt, 20140701-R8F20.txt, 20140701-R10F20.txt, 20150201-R5F1.txt, 20150201-R5F2.txt, 20150201-R5F5.txt, 20150201-R1F5.txt, 20150201-R1F6.txt and 20150201-R1F7.txt available for download from **URL** as input and plots Figure S5.
**MakeFigureS6.m**	This script uses 20140701-R4F16.txt, 20140701-R4F15.txt, 20140701-R4F14.txt, 20140701-R4F13.txt, 20140701-R9F19.txt, 20150201-R4F2.txt, 20150201-R4F3.txt, 20150201-R4F4.txt, 20150201-R4F5.txt and 20150201-R4F6.txt available for download from **URL** as input and plots Figure S6.
**MakeFigureS7.m**	This script uses 20140701-R1F8.txt, 20140701-R1F9.txt, 20140701-R1F10.txt and 20140701-R1F11.txt available for download from **URL** as input and plots Figure S7.
**MakeFigureS8.m**	This script uses 20140701-R1F1.txt, 20140701-R1F2.txt, 20140701-R1F3.txt, 20140701-R1F4.txt, 20140701-R1F5.txt and 20140701-R1F6.txt available for download from **URL** as input and plots Figure S8.
**MakeFigureS9abc.m**	This script uses 20150707-R8F11.txt, 20150707-R9F11.txt, 20150529-R3F19.txt, 20150529-R3F20.txt, 20150529-R4F1.txt, 20150529-R4F2.txt, 20140701-R10F10.txt, 20140701-R8F11.txt, 20140701-R9F9.txt, 20140701-R8F9.txt, 20150201-R4F5.txt, 20150201-R4F6.txt, 20150201-R4F3.txt, 20150201-R4F2.txt, 20140612-R7F6.txt, 20140612-R3F6.txt, 20140612-R5F6.txt and 20140612-R2F6.txt available for download from **URL** as input and plots panels a, b and c of Figure S9.
**MakeFigureS9d.m**	This script uses 20150707-R1F1.txt, 20150707-R10F1.txt, 20150707-R10F18.txt, 20150707-R10F9.txt, 20150529-R2F12.txt, 20150529-R2F18.txt, 20150529-R2F20.txt, 20150529-R1F12.txt, 20150201-R10F6.txt, 20150201-R10F7.txt, 20150201-R10F11.txt, 20150201-R10F18.txt, 20140701-R1F6.txt, 20140701-R1F7.txt, 20140701-R1F18.txt and 20140701-R1F19.txt available for download from **URL** as input and plots panel d of Figure

	S9.
`MakeFigureS10d.m`	This script uses 20150201-R1F8.txt available for download from **URL** as input and plots panel d of Figure S10.
`MakeFigureS10e.m`	This script uses 20150201-R1F8.txt available for download from **URL** as input and plots panel e of Figure S10.
`MakeFigureS10f.m`	This script uses 20150201-R1F8.txt available for download from **URL** as input and plots panel f of Figure S10.
`MakeFigureS12.m`	This script uses NT-TriNuc_filtered_SLN.txt available for download from **URL** as input and plots Figure S12.
`MakeFigureS14abc.m`	This script uses NT-SX4_filtered_SLN.txt available for download from **URL** as input and plots panels a, b and c of Figure S14.
`MakeFigureS15ac.m`	This script uses NT-SX4_filtered_SN.txt available for download from **URL** as input and plots panels a and c of Figure S15.
`MakeFigureS15de.m`	This script uses NT-TriNuc_filtered_SLN.txt available for download from **URL** as input and plots panels d and e of Figure S15.
`paired_end_processing.m`	This is the top-level script for processing deep sequencing data from Illumina.
`fast2aligned.m`	This script parses the FASTQ files, extracts raw sequences, finds reads with mapped barcodes, does alignment and saves the perfect alignments.
`name2librarytype.m`	This script converts an excel sheet of sample names to the type of library and separates samples based on this distinction.
`aligned2unique.m`	This script creates parsed files with untrimmed adapters and saves them to the 'AllParsedFiles' folder. Then it trims the adapters, groups, counts, translates the sequences and saves them to the 'AllUniqueFiles' folder.
`tags2table.m`	This script creates a table file of nucleotide sequences, peptides and frequencies based on a two-character identifier for each individual with samples on this sequencing run. Table files are built from the unique files. Each table file contains all of an individual's samples.
`add.m`	This script adds forward and reverse reads together and converts them to one read.
`alignONEseq.m`	This script uses the aligned files as input, parses sequences and saves them to parsed and table files.
`AlignSeqInline.m`	This script uses sequences with mapped barcodes as input, does alignment and saves the perfect alignments.
`bar2numer.m`	This script converts barcodes to numeric analogs for faster searching.

`cell2numaa.m`	This script converts a string of peptides and their frequencies to a 400*400 array of numbers.
`compareNfiles.m`	This script uses many unique files as input, compares them and saves multisets of sequences and frequencies.
`findM0.m`	This script takes tags, searches for 'M{0}' and returns the row index of those reads with no mismatches between forward and reverse reads.
`findPAR.m`	This script finds the reading parameters for quick reading.
`isperfectmatch.m`	This script finds the perfect alignment for multisets of forward and reverse sequences and returns the corresponding number of mismatches and matches.
`loadNparse.m`	This script reads FASTQ files in chunks of 500,000 sequences (4 lines in a chunk).
`loadNparse2.m`	This script reads aligned files in chunks of 500,000 sequences (6 lines in a chunk).
`makecurves.m`	This script uses multisets of nucleotide sequences as input, calculates the percentage of each codon and plots curves to describe the distribution of 64 codons.
`MakeExpression.m`	This script takes a perfect adapter and creates a random expression that corresponds to perfect, mutated or partial adapters. The output expression is for reads with no mismatches between forward and reverse reads.
`MakemisExpression.m`	This script takes a perfect adapter and creates a random expression that corresponds to mutated adapters. The output expression is for reads with mismatches between forward and reverse reads.
`makeTAG.m`	This script takes an array of tiles, matches, mismatches, forward and reverse reads and creates an array of tags. Each tag contains the length of left and right stuffers, the length of nucleotides in the middle and mismatches positions.
`maketrinuc.m`	This script uses TriNuc codons to make a TriNuc library.
`matchnot0Reads.m`	This script finds library regions in a continuous read with mismatches between forward and reverse reads using predefined library structure and rules for matching this structure.
`matchReads.m`	This script finds library regions in a continuous read using predefined library structure and rules for matching this structure.
`mismatchbars.m`	This script plots the distribution of mismatches at each position for all reads.
`my_str2num.m`	This script converts a string to number.
`nt2aacell.m`	This script converts a cell array or character array of nucleotide sequences to peptide sequences.
`quickRead.m`	The script reads sequences and the corresponding frequencies from the input file and saves them into a string cell array and a double array, respectively.
`quickSave.m`	This script converts sequences or corresponding frequencies into character arrays and saves them.
`rcomplementFAST.m`	This script takes a string array of nucleotide sequences

	and converts them to their corresponding complementary strands.
**readMulticolumn.m**	This script reads table files of nucleotide sequences, peptide sequences and multiple columns of frequencies.
**readMulticolumn1.m**	This script reads table files of peptide sequences, multiple columns of frequencies and other values.
**reads2tileFAST.m**	This script finds alignments for forward and reverse sequences based on the selected tiles.
**reads2tileN.m**	This script finds the optimum tiling for forward and reverse sequences. It then returns the optimum tiling for each forward-reverse read set and numbers of matched base-pairs and mismatched base-pairs at that tiling.
**ReplaceBlankByDash.m**	This script finds all blanks in a string array and converts them to dashes.
**restoreNUC.m**	This script converts a multiset of sequences and frequencies with unique entries to a multiset of sequences with non-unique entries.
**SA.m**	This scripts generates a random sample of frequency vector.
**SQsave.m**	This script finds all reads with non-mapped barcodes and saves them to S_unmatchedbar.txt.
**tag2num.m**	This script takes all transferred tags and converts string to numbers.
**tag_trans.m**	This script transfer tags to new tags to make the positions of mismatches count from the very beginning of the reads and calculates the length of the reads.
**unfiltered2filtered.m**	This script takes a cell array of strings that contains nucleotide sequences and also a filter string, in form of `filter = 'TCT~~~TGT~~~~~~~~~TGTGGTGGAGGT';` where '~' designates any nucleotide and the other nucleotides are part of the filter. It then looks for all nucleotides that are within a specific Hamming distance of the filter (use h=1 for loose filtering and h=0 for strict filtering).
**uniqueCOMB.m**	This script converts a multiset of sequences and frequencies with non-unique entries to a multiset with unique entries only.
**uniquef.m**	This script takes a cell array, finds all unique entries and counts each unique entry.
**visual400x400gen.m**	This script samples a random number of peptides and plots a 20:20 plot.

Table S4. Files used to generate images in specific figures. Files can be found online at http://www.chem.ualberta.ca/~derda/trinucpaper/rawfiles/data/

Figure number	File name	Library type and processing type
Figure 3a	NT-TriNuc_filtered_S10-35cycles.txt	Synthetic NT-TriNuc libraries PCR amplified using 10-35 cycles, sequences not matching SXCX3CGGG were removed.
Figure 3c	NT-TriNuc_filtered_S10-35cycles.txt	Synthetic NT-TriNuc libraries PCR amplified using 10-35 cycles, sequences not matching SXCX3CGGG were removed.
Figure 4a	NT-TriNuc_filtered_SLN.txt	Synthetic, ligated and na ïve NT-TriNuc libraries, sequences not matching SXCX3CGGG were removed.
Figure 4b	NT-TriNuc_filtered_SLN.txt	Synthetic, ligated and na ïve NT-TriNuc libraries, sequences not matching SXCX3CGGG were removed.
Figure4 (d, e, f)	NT-TriNuc_filtered_SLN.txt	Synthetic, ligated and na ïve NT-TriNuc libraries, sequences not matching SXCX3CGGG were removed.
Figure5 (h, i, j)	de_SvsL.txt, de_SvsN.txt and de_LvsN.txt	Differential results of SvsL, SvsN and LvsN.
Figure6 (a, b, c)	NT-SX4_filtered_SN.txt<br>ID-SX4_filtered_N.txt	Synthetic and na ïve NT-SX4 libraries, sequences not matching SX4GGG were removed.<br>Na ïve ID-SX4 libraries, sequences not matching SX4GGG were removed.
Figure7 (d, e, f, g, h, i)	YC_filtered.txt	In the YC_filtered.txt file, YC1-YC4: NT-TriNuc library before modification, ~10^{13} pfu of phage was processed and amplified using 32 cycles of PCR; YC5-YC15: AOB modified NT-TriNuc library captured; YC14-YC18: AOB modified NT-TriNuc library supernatant, ~10^{8} pfu of phage was processed and amplified using 32 cycles of PCR; YC19-YC22: bDCO modified NT-TriNuc library captured; YC23-YC26: bDCO modified NT-TriNuc library supernatant, < 10^{5} pfu of phage was processed and amplified using 32 cycles of PCR. Sequences not matching SXCX3CGGG were removed.
Figure S1 (a, b, c, d, e, f)	NT-TriNuc_filtered_S10-35cycles.txt	Synthetic NT-TriNuc libraries PCR amplified using 10-35 cycles, sequences not matching SXCX3CGGG were removed.
Figure S3 (d)	20150201-R1F8.txt	Synthetic NT-TriNuc library, aligned file contains sequences with and without FR-mismatches
Figure S4 (c, d, e, f)	20150201-R1F8.txt	Synthetic NT-TriNuc library, aligned file contains sequences with and without FR-mismatches
Figure S5	20140701-R1F11.txt	Synthetic NT-TriNuc library
	20140701-R9F20.txt	Ligated NT-TriNuc library
	20140701-R8F20.txt	Na ïve NT-TriNuc library
	20140701-R10F20.txt	Na ïve NT-TriNuc library
	20150201-R5F1.txt	Na ïve NT-TriNuc library
	20150201-R5F2.txt	Na ïve NT-TriNuc library
	20150201-R5F5.txt	Na ïve NT-TriNuc library
	20150201-R1F5.txt	Synthetic NT-TriNuc library
	20150201-R1F6.txt	Synthetic NT-TriNuc library, replicate 2
	20150201-R1F7.txt	Synthetic NT-TriNuc library, replicate 3

Figure S6	20140701-R4F16.txt	Naïve NT-SX4 library
	20140701-R4F15.txt	Naïve NT-SX4 library
	20140701-R4F14.txt	Naïve NT-SX4 library
	20140701-R4F13.txt	Naïve NT-SX4 library
	20140701-R9F19.txt	Ligated NT-SX4 library
	20150201-R4F2.txt	Naïve NT-SX4 library, replicate 1
	20150201-R4F3.txt	Naïve NT-SX4 library, replicate 2
	20150201-R4F4.txt	Naïve NT-SX4 library, replicate 3
	20150201-R4F5.txt	Ligated NT-SX4 library, replicate 1
	20150201-R4F6.txt	Ligated NT-SX4 library, replicate 2
Figure S7	20140701-R1F8.txt	Synthetic NT-SXCX6C library
	20140701-R1F9.txt	Synthetic NT-SXCX5C library
	20140701-R1F10.txt	Synthetic NT-SXCX4C library
	20140701-R1F11.txt	Synthetic NT-TriNuc library
Figure S8	20140701-R1F1.txt	Naïve NT-SX4 library made in bulk
	20140701-R1F2.txt	Naïve NT-SX4 library made in bulk
	20140701-R1F3.txt	Naïve NT-SX4 library made in bulk
	20140701-R1F4.txt	Naïve NT-SX4 library made in bulk
	20140701-R1F5.txt	Naïve NT-SX4 library made in bulk
	20140701-R1F6.txt	Naïve NT-SX4 library made in bulk
Figure S9 (a, b , c, d)	20170707-R1F1.txt	SX7 library
	20170707-R10F1.txt	SX7 library
	20170707-R10F18.txt	SX7 library
	20170707-R10F19.txt	SX7 library
	20150529-R2F12.txt	SX7 library
	20150529-R2F18.txt	SX7 library
	20150529-R2F20.txt	SX7 library
	20150529-R1F12.txt	SX7 library
	20150201-R10F6.txt	SX7 library
	20150201-R10F7.txt	SX7 library
	20150201-R10F11.txt	SX7 library
	20150201-R10F18.txt	SX7 library
	20140701-R1F6.txt	SX7 library
	20140701-R1F7.txt	SX7 library
	20140701-R1F18.txt	SX7 library
	20140701-R1F19.txt	SX7 library
	20150707-R8F11.txt	SX4 library
	20150707-R9F11.txt	SX4 library
	20150529-R3F19.txt	SX4 library
	20150529-R3F20.txt	SX4 library
	20150529-R4F1.txt	SX4 library
	20150529-R4F2.txt	SX4 library
	20140701-R10F10.txt	SX4 library
	20140701-R8F11.txt	SX4 library
	20140701-R9F9.txt	SX4 library
	20140701-R8F9.txt	SX4 library
	20150201-R4F5.txt	SX4 library
	20150201-R4F6.txt	SX4 library
	20150201-R4F3.txt	SX4 library
	20150201-R4F2.txt	SX4 library
	20140612-R7F6.txt	SX4 library
	20140612-R3F6.txt	SX4 library
	20140612-R5F6.txt	SX4 library
	20140612-R2F6.txt	SX4 library

Figure S10<br>(d, e, f)	20150201-R1F8.txt	Synthetic NT-TriNuc library, aligned file contains sequences with and without FR-mismatches
Figure S12	NT-TriNuc_filtered_SLN.txt	Synthetic, ligated and na ïve NT-TriNuc libraries, sequences not matching SXCX3CGGG were removed.
Figure S14<br>(a, b, c)	NT-SX4_filtered_SN.txt	Synthetic and na ïve NT-SX4 libraries, sequences not matching SX4GGG were removed.
Figure S15<br>(a, c, d, e)	TriNuc_filtered_SLN.txt<br><br>NT-SX4_filtered_SN.txt	Synthetic NT-TriNuc libraries PCR-amplified using 25-30 cycles, ligated and na ïve NT-TriNuc libraries, sequences not matching SXCX3CGGG were removed.<br><br>Synthetic and na ïve NT-SX4 libraries, sequences not matching SX4GGG were removed.

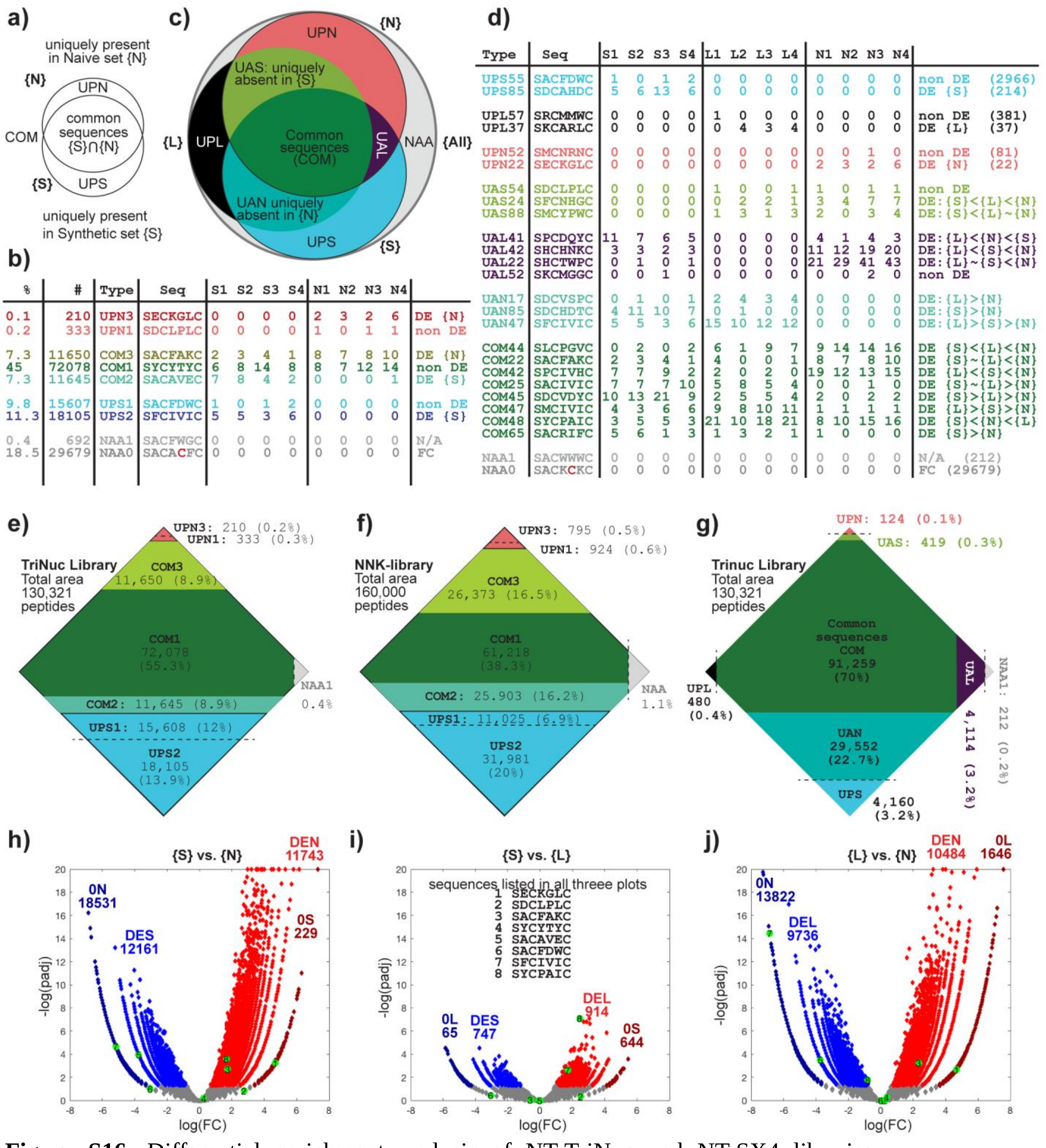

b)

%	#	Type	Seq	S1	S2	S3	S4	N1	N2	N3	N4	
0.1	210	UPN3	SECKGLC	0	0	0	0	2	3	2	6	DE {N}
0.2	333	UPN1	SDCLPLC	0	0	0	0	1	0	1	1	non DE
7.3	11650	COM3	SACFAKC	2	3	4	1	8	7	8	10	DE {N}
45	72078	COM1	SYCYTYC	6	8	14	8	8	7	12	14	non DE
7.3	11645	COM2	SACAVEC	7	8	4	2	0	0	0	1	DE {S}
9.8	15607	UPS1	SACFDWC	1	0	1	2	0	0	0	0	non DE
11.3	18105	UPS2	SFCIVIC	5	5	3	6	0	0	0	0	DE {S}
0.4	692	NAA1	SACFWGC	0	0	0	0	0	0	0	0	N/A
18.5	29679	NAA0	SACACFC	0	0	0	0	0	0	0	0	FC

d)

Type	Seq	S1	S2	S3	S4	L1	L2	L3	L4	N1	N2	N3	N4	
UPS55	SACFDWC	1	0	1	2	0	0	0	0	0	0	0	0	non DE (2966)
UPS85	SDCAHDC	5	6	13	6	0	0	0	0	0	0	0	0	DE {S} (214)
UPL57	SRCMMWC	0	0	0	0	1	0	0	0	0	0	0	0	non DE (381)
UPL37	SKCARLC	0	0	0	0	0	4	3	4	0	0	0	0	DE {L} (37)
UPN52	SMCNRNC	0	0	0	0	0	0	0	0	0	0	1	0	non DE (81)
UPN22	SECKGLC	0	0	0	0	0	0	0	0	2	3	2	6	DE {N} (22)
UAS54	SDCLPLC	0	0	0	0	1	0	0	1	1	0	1	1	non DE
UAS24	SFCNHGC	0	0	0	0	0	2	2	1	3	4	7	7	DE:{S}<{L}<{N}
UAS88	SMCYPWC	0	0	0	0	1	3	1	3	2	0	3	4	DE:{S}<{L}~{N}
UAL41	SPCDQYC	11	7	6	5	0	0	0	0	4	1	4	3	DE:{L}<{N}<{S}
UAL42	SHCHNKC	3	3	2	3	0	0	0	0	11	12	19	20	DE:{L}<{S}<{N}
UAL22	SHCTWPC	0	1	0	1	0	0	0	0	21	29	41	43	DE:{L}~{S}<{N}
UAL52	SKCMGGC	0	0	1	0	0	0	0	0	0	0	2	0	non DE
UAN17	SDCVSPC	0	1	0	1	2	4	3	4	0	0	0	0	DE:{L}>{N}
UAN85	SDCHDTC	4	11	10	7	0	1	0	0	0	0	0	0	DE:{S}>{N}
UAN47	SFCIVIC	5	5	3	6	15	10	12	12	0	0	0	0	DE:{L}>{S}>{N}
COM44	SLCPGVC	0	2	0	2	6	1	9	7	9	14	14	16	DE {S}<{L}<{N}
COM22	SACFAKC	2	3	4	1	4	0	0	1	8	7	8	10	DE {S}~{L}<{N}
COM42	SPCIVHC	7	7	9	2	2	0	2	0	19	12	13	15	DE {L}<{S}<{N}
COM25	SACIVIC	7	7	7	10	5	8	5	4	0	0	1	0	DE {S}~{L}>{N}
COM45	SDCVDYC	10	13	21	9	2	5	5	4	2	0	2	2	DE {S}>{L}>{N}
COM47	SMCIVIC	4	3	6	6	9	8	10	11	1	1	1	1	DE {L}>{S}>{N}
COM48	SYCPAIC	3	5	5	3	21	10	18	21	8	10	15	16	DE {S}<{N}<{L}
COM65	SACRIFC	5	6	1	3	1	3	2	1	1	0	0	0	DE {S}>{N}
NAA1	SACWWWC	0	0	0	0	0	0	0	0	0	0	0	0	N/A (212)
NAA0	SACKCKC	0	0	0	0	0	0	0	0	0	0	0	0	FC (29679)

Figure S16. Differential enrichment analysis of NT-TriNuc and NT-SX4 libraries. Theoretically, there are 19^4 = 130,321 and 20^4 = 160,000 unique sequences in synthetic, ligated and naïve NT-TriNuc and NT-NNK libraries (sets), respectively. (**a**) Venn Diagram comparison of Naïve {**N**} and Synthetic {**S**} sets and definitions of

common (**COM**) and uniquely present sequences (**UPS**, **UPN**). (**b**) Table of sequences and their copy numbers observed in sequencing highlights that sequences from **COM**, **UPS**, **UPN** subsets can be differentially enriched (**DE**) and **non-DE**. Both synthetic and naïve libraries have been sequenced four times, thus, {**S**} and {**N**} sets have four replicates. Abbreviations are as follows:

UPN1: sequence is uniquely present in Naïve library but not in Synthetic, however, the difference between the copy number of this sequence in Naïve and Synthetic is not significant (i.e., non-DE)

UPN3: sequence is uniquely present in Naïve library but not in Synthetic and the difference between the copy number of this sequence in Naïve and Synthetic is significant (i.e., DE) at a predefined significance level ($p<0.05$)

COM1: sequence is common in Synthetic and Naïve library and the copy number of this sequence in Naïve and Synthetic is not significant.

COM2: sequence is common in Synthetic and Naïve library but the copy number of this sequence is significantly higher in Synthetic Library

COM3: sequence is common in Synthetic and Naïve library but the copy number of this sequence is significantly higher in Naïve library

UPS1, UPS2: uniquely present in synthetic library and the difference in copy number between Naïve and Synthetic library is either insignificant (1) or significant (2).

NAA1: not available in any sets (neither present in {**S**} nor {**N**} sets) but theoretically possible.

NAA0: not available in any sets (neither present in {**S**} nor {**N**} sets) and theoretically not plausible (contains forbidden codons).

(**c**) Comparison of {**S**}, {**N**} and ligated {**L**} sets, and definition of "uniquely absent" sets UAN, UAS, UAL. Sequence that is uniquely absent from {**N**} set but present in {**L**} and {**S**} sets belongs to UAN set. etc. (**d**) Example of reads, their copy numbers and their classifications; there exist several DE-classes: see Figure S13 for detailed description of all classes. As each sample was sequenced 4 times, there are four replicates of synthetic (S1, S2, S3, S4), ligated (L1, L2, L3 and L4) and na ïve (N1, N2, N3 and N4) libraries. (**e**) To-scale representation of the entire NT-TriNuc library, in which the area of the square represents 19^4=130,321 unique peptides and the area of each segment is proportional to the number of unique sequences in each type listed in (**b**). For example, there are 95,373 (**COM1**: 72,078, **COM2**: 11,645, **COM3**: 11,650) unique sequences present both in {**S**} and {**N**} sets. (**f**) Analogous description of NT-NNK library. 46-55% of the library contains sequences that are neither enriched nor depleted between {**S**} and {**N**} sets. Up to 30% of library sequences are uniquely present in {**S**}. (**g**) Analogous to-scale representation of an overlay of {**S**} and {**L**} and {**N**} from NT-TriNuc library shows that of 26% of sequences identified as UPN in (**e**), 22% are present in both {**S**} and {**L**} (i.e., "Uniquely Absent from Naïve" or UAN) and only 3.2% and 0.4% are unique to {**S**} or {**L**}. Note that in the **COM** set in (**g**), DE-information is omitted for clarity. (**h-j**) Volcano plots describing DE-comparison of {**S**}, {**N**} and {**L**}. {**S**} and {**L**} are most similar to one another whereas {**N**} is different from both {**S**} and {**L**}. Each type of sequences listed in (**b**) is mapped on each volcano plot. Abbreviations: **DES** – differentially enriched in synthetic, **DEL** – differentially enriched in ligated, **DEN** – differentially enriched in na ïve, **0S** – not present in synthetic, **0L** – not present in ligated, **0N** – not present in na ïve. For details and algorithm for DE-analysis see R.zip file in the Supplementary Information for *.RMD R-code.
