# Supplementary material for: Compositional Bias in Naïve and Chemically-modified Phage-Displayed Libraries uncovered by Paired-end Deep Sequencing
Source: Sci Rep. 2018 Jan 19;8:1214. doi: 10.1038/s41598-018-19439-2 (PMC5775325; doi:10.1038/s41598-018-19439-2)
Supplement: Supplementary file 3 — Supplementary Information [file 41598_2018_19439_MOESM3_ESM.zip › R/NT-TriNuc_diffAnalysis_SLN.pdf]

Analysis of phage data: Compositional Bias in Naïve and Chemically-modified Phage-Displayed Libraries uncovered by Paired-end Deep Sequencing

Andrea

June 08 2017

This document presents the full source code used to obtain results for the differential analysis of the phage data for the Bifang He et al. 2017 paper. Specifically, the Bioconductor package edgeR is used to fit a negative binomial model to each sequence and to implement a quasi-likelihood test to identify differentially enriched sequences in the (synthetic DNA, DNA ligated into the cloning vector and naïve) libraries.

This new version of the analysis removes the filtering step for low counts, and writes out more complete information in the output files (i.e., normalized counts).

Load packages, data, and annotation

We begin by loading the necessary packages for the data analysis, as well as the formatted data themselves. Note that the main package to be used for the differential analysis is edgeR.

```
## Set working directory, load libraries
library(edgeR)

## Loading required package: limma

library(RColorBrewer)

rerun <- TRUE

if(rerun == TRUE) {
  ## Read full data and format
  full.dat <- read.table("NT-TriNuc_filtered_SLN_pep.txt", fill=TRUE,
                         stringsAsFactors=FALSE)
  colnames(full.dat) <- full.dat[1,]

  ## Remove the title line
  full.dat <- full.dat[-c(1,2),]

  ## Convert counts to numeric, identify conditions via column labels
  pep.dat <- sapply(full.dat[,-c(1)], as.numeric)
  rownames(pep.dat) <- full.dat[,1]
```

```
  colnames(pep.dat) <- paste(substr(colnames(full.dat)[2:13],1,1),'-',
substr(colnames(full.dat)[2:13],2,2), sep="")
  colnames(pep.dat) <- chartr(".", "-", colnames(pep.dat))

  dat<- pep.dat

  dim(dat)

  conds <- factor(substr(unlist(lapply(strsplit(colnames(dat), "-"), fu
nction(x) x[1])), 1, 3))
  conds

}

##  [1] S S S S L L L L N N N N
## Levels: L N S

if(rerun == FALSE) {
  load("NT-TriNuc_diffAnalysis_SLN.RData")
}

dim(dat)

## [1] 160000     12

# quantile(dat)
barplot(colSums(dat), col = c(rep("grey50", 4), rep("grey90", 4), rep("
grey50", 4)), ylab = "Library sizes", main="")
```

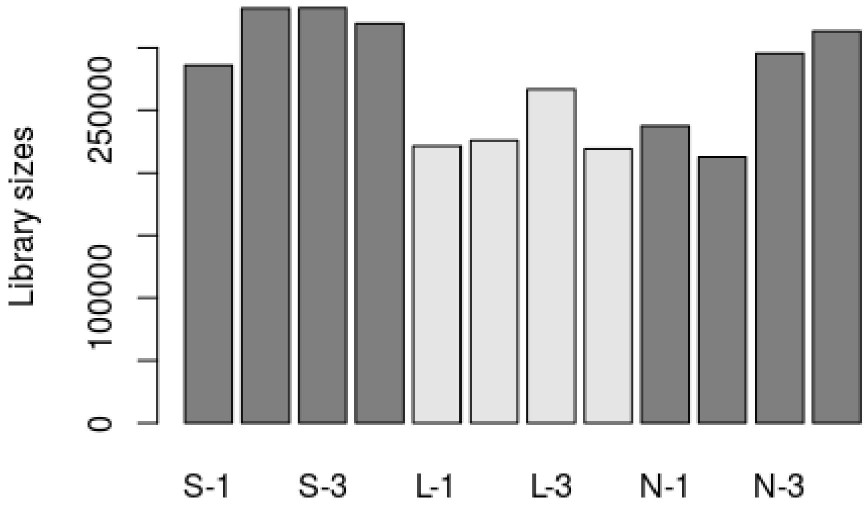

edgeR analysis (with TMM normalization)

In this section, we make use of the Bioconductor package edgeR (Robinson and Smyth, 2008; Robinson and Smyth, 2007; Robinson et al., 2010) to fit a negative binomial model to each sequence and to implement a quasi-likelihood F test (Lund et al., 2012) for differential expression. Note that the quasi-likelihood F test provides more robust and reliable error rate control than the standard likelihood ratio test when the number of replicates is small. The novelty of the edgeR package is that it implements an empirical Bayes method to estimate sequence-specific biological variation (i.e., overdispersion). In particular, rather than using an overall, common estimate for dispersion parameters or a per-sequence dispersion parameter estimate, edgeR calculates moderated dispersion parameter estimates that are reliable even for small samples. These moderated dispersion parameters allow information to be shared between sequences while still maintaining sequence-specific dispersion estimates by squeezing tagwise dispersions toward the common dispersion.

Within the edgeR framework, we make use of the Trimmed Mean of M-values (TMM) normalization (Robinson and Oshlack, 2010) to account for differences in library composition among the different libraries. This technique find a set of scaling factors for library sizes (yielding so-called effective library sizes) that minimize log-fold changes between the samples for most sequences.

We next examine sequences for which reads are observed for a single group (and 0's are observed for the remaining two groups) and for which reads are uniquely absent from one group (and none zeros are observed for the remaining two groups).

For the remaining analysis, we additionally examine sequences with less than 3 counts per million (CPM) in at least 4 samples, which is a common filter used in edgeR differential analyses for very weakly expressed sequences that are unlikely to be detected as differentially expressed. However, in our analysis this step only serves to examine those sequences that have few reads; note that with the exception of sequences that are uniquely observed in a single group and sequences that are uniquely absent from one group (as noted above), we retain these weakly abundant sequences for the subsequent differential analysis. The full set of weakly expressed sequences (i.e., those with < 3 CPM in at least 4 samples) is saved in the file `low_count_sequences.txt`. In addition, the subset of sequences that are uniquely observed in a single group are saved in the file `count_sequences_uniqueGroup.txt`. The subset of sequences that are uniquely absent from one group are save in the file `count_sequences_uniqueabsent.txt`.

Finally, we fit a negative binomial model such that

$$\log(\mu_{ij}/S_j) = X_j\beta_i = \beta_{0i} + \beta_{1i}I_{j,L} + \beta_{2i}I_{j,N}$$

where μ_{ij} represents the mean of sequence i in sample j, and $I_{j,N}$ is the indicator function for sample j belonging to group N and so on.

We provide a multidimensional scaling (MDS) plot below, which indicates that a good separation is seen among the three library types. We also provide a plot of the (raw, trended, and squeezed) quasi-likelihood estimates of dispersion estimated using edgeR; everything looks good with this plot.

After running edgeR with a Benjamini-Hochberg correction to control the false discovery rate (FDR) at $\alpha = 0.05$, see summaries below for the numbers of differentially enriched sequences.

```
if(rerun == TRUE) {

  conds <- relevel(conds, "S")
  design <- model.matrix(~0+conds)
  ## S = synthetic, L = ligated, N = naïve,
  colnames(design) <- c("synthetic", "ligated", "naïve")
  DGE <- DGEList(counts=dat, group=conds)

  ## Normalize with TMM
  DGE <- calcNormFactors(DGE)
  DGE$samples



  DGE_sums <- t(rowsum(t(DGE$counts), group=as.vector(conds)))
```

```
  ## First, we check sequences that are not available in synthetic or n
aïve libraries, but do not remove them from differential enrichment ana
lysis
  NAA <- (which(DGE_sums[,"N"] == 0 & DGE_sums[,"S"] == 0 & DGE_sums[,"
L"] == 0))
  cat("\nTotal unique peptides not available in all groups:", length(NA
A),"\n")
  cat("\nTotal unique peptides not available in all groups:", length(NA
A),
      "(", 100*round(length(NAA)/nrow(DGE), 3),
      "% of sequences )\n")

  DGE_NAA <- DGE[NAA,]
  dim(DGE_NAA)
  write.table(DGE_NAA$counts, "count_sequences_NAA.txt",
              col.names=TRUE, quote=FALSE)

  ## Then, we check sequences that are uniquely present in one group an
d uniquely absent from one group, but do not remove them
  N_pep  <- (which(DGE_sums[,"N"] > 0))
  N_only <- (which(DGE_sums[,"N"] > 0 &
                         rowSums(DGE_sums[,-which(colnames(DGE_sums)
== "N")])==0))
  N_absent <- (which(DGE_sums[,"N"] == 0 & DGE_sums[,"S"]>0 & DGE_sums[,
"L"]>0))

  L_pep <- (which(DGE_sums[,"L"] > 0))
  L_only <- (which(DGE_sums[,"L"] > 0 &
                         rowSums(DGE_sums[,-which(colnames(DGE_sums)
== "L")])==0))
  L_absent <- (which(DGE_sums[,"L"] == 0 & DGE_sums[,"S"]>0 & DGE_sums[,
"N"]>0))

  S_pep <- (which(DGE_sums[,"S"] > 0))
  S_only <- (which(DGE_sums[,"S"] > 0 &
                         rowSums(DGE_sums[,-which(colnames(DGE_sums)
== "S")])==0))
  S_absent <- (which(DGE_sums[,"S"] == 0 & DGE_sums[,"L"]>0 & DGE_sums[,
"N"]>0))

  cat("\nTotal unique peptides in naïve:", length(N_pep),
      "\nTotal unique peptides in ligated:", length(L_pep),
      "\nTotal unique peptides in synthetic:", length(S_pep), "\n")

  cat("\nUniquely present in naïve:", length(N_only),
      "\nUniquely present in ligated:", length(L_only),
      "\nUniquely present in synthetic:", length(S_only), "\n")
  cat("Total unique to one group:", sum(c(length(N_only),
```

```
                                                        length(L_only), length(S_onl
y))),
      "(", 100*round(sum(c(length(N_only), length(L_only),
                             length(S_only)))/nrow(DGE), 3),
      "% of sequences )\n")

  cat("\nUniquely absent naïve:", length(N_absent),
      "\nUniquely absent ligated:", length(L_absent),
      "\nUniquely absent synthetic:", length(S_absent), "\n")
  cat("Total uniquely absent from one group:", sum(c(length(N_absent),
                                                        length(L_absent), length(S_ab
sent))),
      "(", 100*round(sum(c(length(N_absent), length(L_absent),
                             length(S_absent)))/nrow(DGE), 3),
      "% of sequences )\n")

  DGE_unique <- DGE[c(N_only, L_only, S_only),]
  dim(DGE_unique)
  write.table(DGE_unique$counts, "count_sequences_uniqueGroup.txt",
              col.names=TRUE, quote=FALSE)
  write.table(DGE[S_only,]$counts, "count_sequences_UPS.txt", col.names
=TRUE, quote=FALSE)
  write.table(DGE[L_only,]$counts, "count_sequences_UPL.txt", col.names
=TRUE, quote=FALSE)
  write.table(DGE[N_only,]$counts, "count_sequences_UPN.txt", col.names
=TRUE, quote=FALSE)


  DGE_absent <- DGE[c(N_absent, L_absent, S_absent),]
  dim(DGE_absent)
  write.table(DGE_absent$counts, "count_sequences_uniqueabsent.txt",
              col.names=TRUE, quote=FALSE)
  write.table(DGE[S_absent,]$counts, "count_sequences_UAS.txt", col.nam
es=TRUE, quote=FALSE)
  write.table(DGE[L_absent,]$counts, "count_sequences_UAL.txt", col.nam
es=TRUE, quote=FALSE)
  write.table(DGE[N_absent,]$counts, "count_sequences_UAN.txt", col.nam
es=TRUE, quote=FALSE)

  DGE_all <- DGE
  dim(DGE_all)


  ## Examine weakly abundant sequences, but do not remove them
  keep <- rowSums(cpm(DGE_all)>1) >= 3
  table(keep)
  DGE_low <- DGE_all[which(keep == FALSE), , keep.lib.sizes=FALSE]
  dim(DGE_low$counts)
  nrow(DGE_low) / nrow(DGE_all)
```

```
  write.table(DGE_low$counts, "low_count_sequences.txt", col.names=TRUE,
 quote=FALSE)


  ## Estimate dispersion parameters, fit quasi-likelihood, plot dispers
ions
  DGE_all <- estimateDisp(DGE_all, design, robust=TRUE)
  fit <- glmQLFit(DGE_all, design, robust=TRUE)

  ## Ligated versus synthetic
  cn <- makeContrasts(synthetic - ligated, levels=design)
  qlf_LvsS <- glmQLFTest(fit, contrast=cn)
  summary(decideTestsDGE(qlf_LvsS, adjust.method="BH"))
  de_LvsS.all <- data.frame(sequence=rownames(DGE_all$counts),
                            DGE_all$counts, qlf_LvsS$table,
                           padj = p.adjust(qlf_LvsS$table$PValue, metho
d="BH"))
  o <- order(de_LvsS.all$padj)
  de_LvsS.all <- de_LvsS.all[o,]
  write.table(de_LvsS.all,
              "de_SvsL.txt", quote=FALSE, sep="\t", row.names=FALSE)


  ## Synthetic versus naïve
  cn <- makeContrasts(synthetic - naïve, levels=design)
  qlf_NvsS <- glmQLFTest(fit, contrast=cn)
  summary(decideTestsDGE(qlf_NvsS, adjust.method="BH"))
  de_NvsS.all <- data.frame(sequence=rownames(DGE_all$counts),
                            DGE_all$counts, qlf_NvsS$table,
                           padj = p.adjust(qlf_NvsS$table$PValue, metho
d="BH"))
  o <- order(de_NvsS.all$padj)
  de_NvsS.all <- de_NvsS.all[o,]
  write.table(de_NvsS.all, "de_SvsN.txt",
              quote=FALSE, sep ="\t", row.names=FALSE)

  ## naïve versus ligated
  cn <- makeContrasts(ligated - naïve, levels=design)
  qlf_NvsL <- glmQLFTest(fit, contrast=cn)
  summary(decideTestsDGE(qlf_NvsL, adjust.method="BH"))
  de_NvsL.all <- data.frame(sequence=rownames(DGE_all$counts),
                            DGE_all$counts, qlf_NvsL$table,
                           padj = p.adjust(qlf_NvsL$table$PValue, metho
d="BH"))
  o <- order(de_NvsL.all$padj)
  de_NvsL.all <- de_NvsL.all[o,]

  write.table(de_NvsL.all, "de_LvsN.txt",
              quote=FALSE, sep ="\t", row.names=FALSE)
```

```
  save.image("NT-TriNuc_diffAnalysis_SLN.RData")
}
```

```
##
## Total unique peptides not available in all groups: 29891
##
## Total unique peptides not available in all groups: 29891 ( 18.7 % of
 sequences )
##
## Total unique peptides in naïve: 95916
## Total unique peptides in ligated: 121711
## Total unique peptides in synthetic: 129086
##
## Uniquely present in naïve: 124
## Uniquely present in ligated: 480
## Uniquely present in synthetic: 4160
## Total unique to one group: 4764 ( 3 % of sequences )
##
## Uniquely absent naïve: 29553
## Uniquely absent ligated: 4114
## Uniquely absent synthetic: 419
## Total uniquely absent from one group: 34086 ( 21.3 % of sequences )
```

```
## MDS plot
group_color <- brewer.pal(3, "Dark2")
plotMDS(DGE_all, col=group_color[apply(model.matrix(~0+conds), 1, which.
max)])
legend("topright", legend=levels(conds), pch=19, col=group_color, ncol=
2)
```

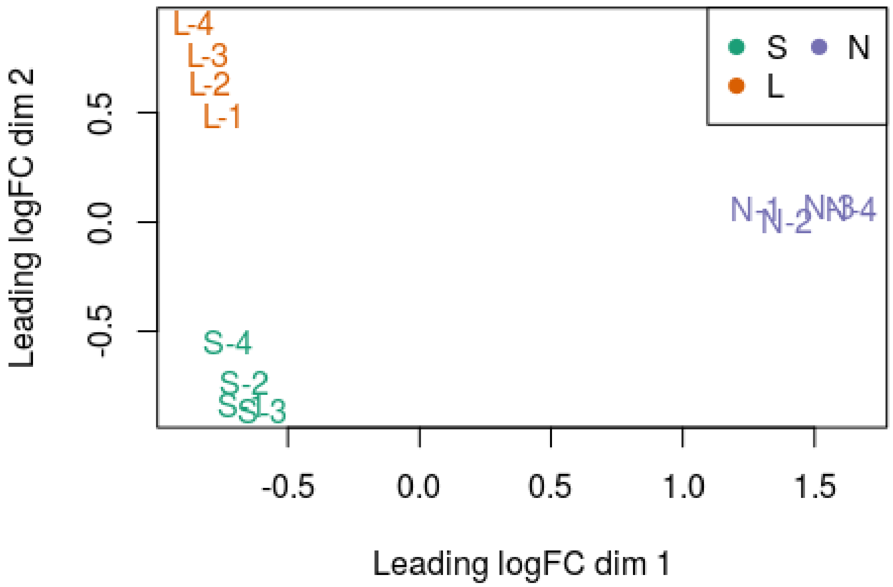


```r
## Quasi-likelihood dispersion plot
plotQLDisp(fit)
```

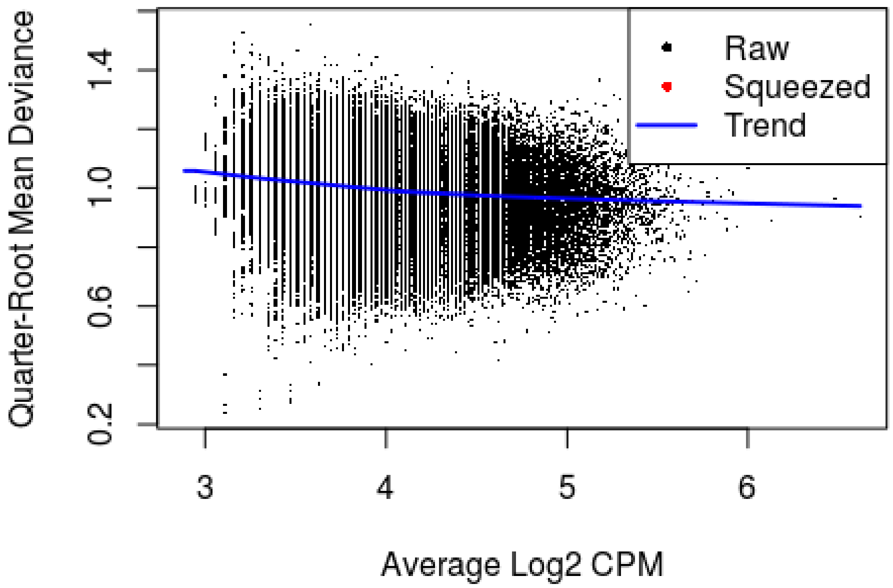

We examine the histograms of raw p-values for each of the three contrasts; in all cases, we note roughly uniform distributions of p-values between 0 and 1 (corresponding to the expected distribution of p-values under the null hypothesis), with peaks occasionally observed close to 0 (corresponding to sequences under the alternative hypothesis of differential abundance) or close to 1 (corresponding to weak counts) -- basically, this means that things look more or less good and there do not seem to be major problems with the model fit by edgeR.

```
par(mfrow=c(1,3))
hist(qlf_LvsS$table$PValue, main = "LvsS", breaks=10, col="grey", xlab=
"Raw p-values")
hist(qlf_NvsS$table$PValue, main = "NvsS", breaks=10, col="grey", xlab=
"Raw p-values")
hist(qlf_NvsL$table$PValue, main = "NvsL", breaks=10, col="grey", xlab=
"Raw p-values")
```

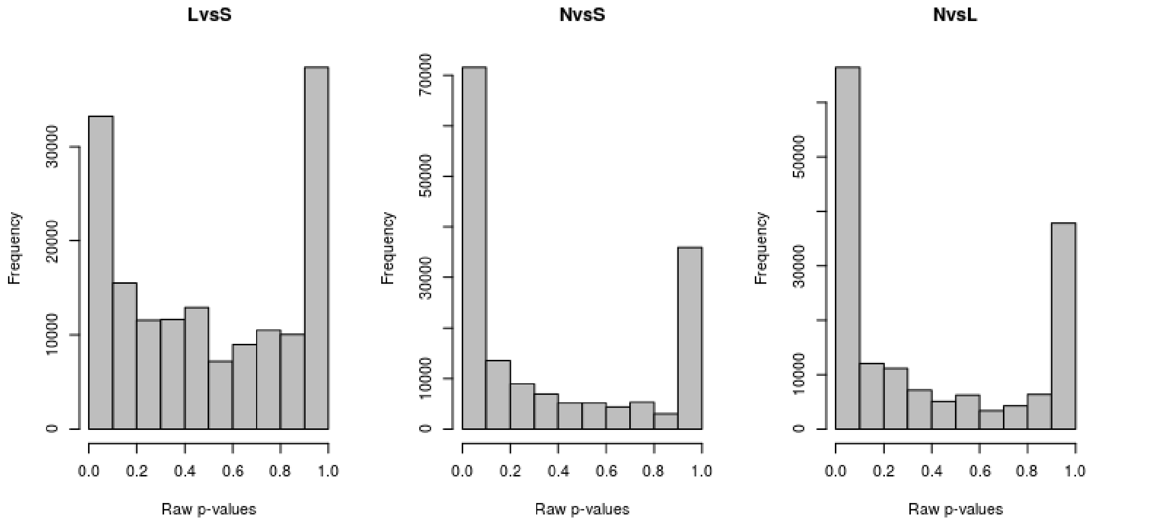

Finally, we examine the trends of results for the differentially enriched sequences. We note that only 2370 unique sequences are differentially enriched between the ligated and synthetic libraries, while a large number of reads are differentially enriched between the ligated and naïve libraries (23558 over-enriched and 12130 under-enriched) and the synthetic and naïve libraries (30692 over-enriched and 11972 under-enriched). Finally, 35688 and 42664 differentially enriched sequences are found for the naïve versus ligated and naïve versus synthetic compairsons (see Venn diagram below).

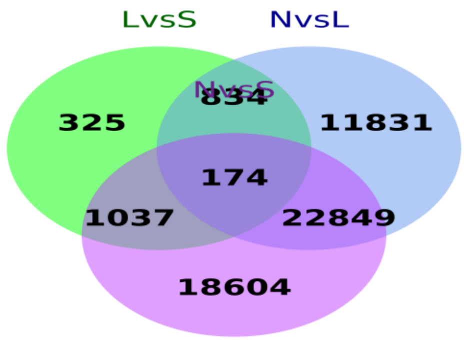


```
library(VennDiagram)

## Loading required package: grid

## Loading required package: futile.logger

v <- suppressWarnings(venn.diagram(list(
                       LvsS=which(decideTestsDGE(qlf_LvsS, adjust.metho
d="BH")!=0),
                       NvsL=which(decideTestsDGE(qlf_NvsL, adjust.metho
d="BH")!=0),
                       NvsS=which(decideTestsDGE(qlf_NvsS, adjust.metho
d="BH")!=0)),
                       filename="venn.png",
                  col = "transparent",
                  fill = c("green", "cornflowerblue", "darkorchid1"),
                  alpha = 0.50,
                  cex = 1.5,
                  fontfamily = "sansserif",
                  fontface = "bold",
                  cat.col = c("darkgreen", "darkblue", "darkorchid4"),
                  cat.cex = 1.5,
                  cat.pos = 0,
                  cat.dist = 0.07,
                  cat.fontfamily = "sansserif",
                  margin = 0.2))
```

Finally, we summarized uniquely present peptides, uniquely absent peptides and sequences not available at all groups. The number of unqiue peptides in each class is shown in the following table.

Upep	UAS	UAL	UAN	UPS	UPL	UPN	NAA
Number	419	4114	29553	4160	480	124	29891

We also summarized the differentially enriched peptides. Note that S = synthetic, L = ligated, N = naïve. The table should be read as follows: using the second line as an example, in comparing the ligated versus naïve libraries (L - N), 23558 (12130) sequences were identified as differentially over-abundant (under-abundant) in the ligated libraries.

Comparison	DE sequences (edgeR)
S - L	1391 (979)
L - N	23558 (12130)
S - N	30692 (11972)

Session Info

```
sessionInfo()

## R version 3.4.0 (2017-04-21)
## Platform: x86_64-pc-linux-gnu (64-bit)
## Running under: Ubuntu 16.04.1 LTS
##
## Matrix products: default
## BLAS: /usr/lib/libblas/libblas.so.3.6.0
## LAPACK: /usr/lib/lapack/liblapack.so.3.6.0
##
## locale:
##  [1] LC_CTYPE=en_CA.UTF-8       LC_NUMERIC=C
##  [3] LC_TIME=en_CA.UTF-8        LC_COLLATE=en_CA.UTF-8
##  [5] LC_MONETARY=en_CA.UTF-8    LC_MESSAGES=en_CA.UTF-8
##  [7] LC_PAPER=en_CA.UTF-8       LC_NAME=C
##  [9] LC_ADDRESS=C               LC_TELEPHONE=C
## [11] LC_MEASUREMENT=en_CA.UTF-8 LC_IDENTIFICATION=C
##
## attached base packages:
## [1] grid      stats     graphics  grDevices utils     datasets  meth
ods
## [8] base
##
## other attached packages:
## [1] VennDiagram_1.6.17  futile.logger_1.4.3 RColorBrewer_1.1-2
## [4] edgeR_3.17.10       limma_3.31.22
##
## loaded via a namespace (and not attached):
##  [1] locfit_1.5-9.1       Rcpp_0.12.10         codetools_0.2-15
##  [4] lattice_0.20-35      digest_0.6.12        rprojroot_1.2
##  [7] futile.options_1.0.0 backports_1.0.5      magrittr_1.5
## [10] evaluate_0.10        stringi_1.1.5        rmarkdown_1.4
## [13] splines_3.4.0        statmod_1.4.29       lambda.r_1.1.9
## [16] tools_3.4.0          stringr_1.2.0        yaml_2.1.14
## [19] compiler_3.4.0       htmltools_0.3.5      knitr_1.15.1
```

References

-- Robinson, MD, and Smyth, GK (2008). Small sample estimation of negative binomial dis- persion, with applications to SAGE data. Biostatistics 9, 321-332.

-- Robinson, MD, and Smyth, GK (2007). Moderated statistical tests for assessing differences in tag abundance. Bioinformatics 23, 2881-2887.

-- Robinson, MD, McCarthy, DJ, Smyth, GK (2010). edgeR: a Bioconductor package for differential expression analysis of digital gene expression data. Bioinformatics 26, 139-140.

-- Robinson, MD, and Oshlack, A (2010). A scaling normalization method for differential expression analysis of RNA-seq data. Genome Biology 11, R25.

-- Lund, SP, Nettleton, D, McCarthy, DJ, Smyth, GK (2012). Detecting differential expression in RNA-sequence data using quasi-likelihood with shrunken dispersion estimates. Statistical Applications in Genetics and Molecular Biology Volume 11, Issue 5, Article 8.
